# Association of plasma trace element levels with neovascular age-related macular degeneration

**DOI:** 10.1016/j.exer.2020.108324

**Published:** 2020-12

**Authors:** Thomas J. Heesterbeek, Mansour Rouhi-Parkouhi, Stephanie J. Church, Yara T. Lechanteur, Laura Lorés-Motta, Nikolaos Kouvatsos, Simon J. Clark, Paul N. Bishop, Carel B. Hoyng, Anneke I. den Hollander, Richard D. Unwin, Anthony J. Day

**Affiliations:** a Department of Ophthalmology, Donders Institute for Brain, Cognition and Behaviour, Radboud University Medical Center, Nijmegen, the Netherlands; b Wellcome Trust Centre for Cell-Matrix Research, Division of Cell-Matrix Biology & Regenerative Medicine, School of Biological Sciences, Faculty of Biology, Medicine and Health, University of Manchester, Manchester Academic Health Science Centre, Oxford Road, Manchester, M13 9PT, UK; c Lydia Becker Institute of Immunology and Inflammation, Faculty of Biology, Medicine and Health, University of Manchester, Manchester Academic Health Science Centre, Oxford Road, Manchester, M13 9PT, UK; d Division of Cardiovascular Sciences, School of Medical Sciences, Faculty of Biology Medicine and Health, The University of Manchester, Core Technology Facility, Grafton Street, Manchester, M13 9NT, UK; e Department of Human Genetics, Donders Institute for Brain, Cognition and Behaviour, Radboud University Medical Center, Nijmegen, the Netherlands; f Division of Evolution and Genomic Sciences, School of Biological Sciences, Faculty of Biology, Medicine and Health, University of Manchester, Manchester, M13 9PT, UK; g Institute for Ophthalmic Research, Eberhard Karls University of Tübingen, Elfriede-Aulhorn-Straße 7, 72076, Tübingen, Germany; h Manchester Royal Eye Hospital, Manchester University NHS Foundation Trust, Manchester Academic Health Sciences Centre, Manchester M13 9WL, UK; i Stoller Biomarker Discovery Centre and Division of Cancer Sciences, School of Medical Sciences, Faculty of Biology Medicine and Health, The University of Manchester, CityLabs 1.0 (3rd Floor), Nelson Street, Manchester, M13 9NQ, UK

**Keywords:** Age-related macular degeneration, Trace elements, Plasma, Barium, Cadmium, Chromium, Al, aluminum, AMD, age-related macular degeneration, ANOVA, analysis of variance, As, arsenic, Ba, barium, Bi, bismuth, BM, bruch's membrane, BMI, body mass index, Ca, calcium, Cd, cadmium, Ce, cerium, CFP, color fundus photographs, CIRCL, Cologne Image Reading Center and Laboratory, Co, cobalt, Cr, chromium, Cu, copper, EUGENDA, European Genetic Database, ETDRS, Early Treatment Diabetic Retinopathy Study, FA, fluorescein angiography, FDR, false discovery rate, Fe, iron, GA, geographic atrophy, Ge, germanium, He, helium, I-AS, Agilent Integrated autosampler, ICGA, indocyanine green angiography, ICP-MS, inductively coupled plasma mass spectrometry, IQR, interquartile range, In, indium, K, potassium, Li, lithium-6, LOCS, lens opacity classification system, LOQ, limit of quantification, MDA, malondialdehyde, Mg, magnesium, Mn, manganese, Mo, molybdenum, Na, sodium, nAMD, neovascular age-related macular degeneration, Ni, nickel, NIST, National Institute of Standards and Technology, NSAIDs, Nonsteroidal anti-inflammatory drugs, OCT, optical coherence tomography, OR, odds ratio, Pb, lead, ROS, reactive oxygen species, RPE, retinal pigment epithelium, Sb, antimony, Sc, scandium, SD, standard deviation, Se, selenium, smMIPs, single molecule molecular inversion probes, SRM, Standard Reference Material, Tb, terbium, Tl, thallium, V, vanadium, VEGF, vascular endothelial growth factor, Y, yttrium, Zn, zinc

## Abstract

Although the triggers causing angiogenesis in the context of neovascular age-related macular degeneration (nAMD) are not fully understood, oxidative stress is likely involved. Oxidative stress in the eye can occur through exposure of macular tissues to sunlight and local or systemic exposure to oxidative stressors associated with environmental or lifestyle factors. Because trace elements have been implicated as regulators of oxidative stress and cellular antioxidant defense mechanisms, we hypothesized that they may play a role as a risk factor, modifying the progression toward nAMD.

Herein, we determined whether levels of human plasma trace elements are different in 236 individuals with nAMD compared to 236 age-matched controls without AMD. Plasma levels of 16 trace elements including arsenic, barium, calcium, cadmium, cobalt, chromium, copper, iron, magnesium, manganese, molybdenum, lead, antimony, selenium, vanadium and zinc were measured using inductively coupled plasma mass spectrometry. Associations of trace elements with demographic, environmental and lifestyle factors and AMD-associated genetic variants were assessed.

Elevated levels of barium and cadmium and reduced levels of chromium were observed in nAMD patients compared to controls. Mean plasma concentrations of barium were 1.35 μg/L (standard deviation [SD] 0.71) in nAMD and 1.15 μg/L (SD 0.63) in controls (P = 0.001). Mean levels of chromium were 0.37 μg/L (SD 0.22) in nAMD and 0.46 μg/L (SD 0.34) in controls (P = 0.001). Median levels for cadmium, which were not normally distributed, were 0.016 μg/L (interquartile range [IQR] 0.001–0.026) in nAMD and 0.012 μg/L (IQR 0.001–0.022) in controls (P = 0.002). Comparison of the Spearman's correlation coefficients between nAMD patients and controls identified a difference in correlations for 8 trace elements. Cadmium levels were associated with the smoking status (P < 0.001), while barium levels showed a trend of association with the usage of antihypertensive drugs. None of the AMD-associated genetic variants were associated with any trace element levels.

In conclusion, in this case-control study we detected elevated plasma levels of barium and cadmium and reduced plasma levels of chromium in nAMD patients. An imbalance in plasma trace elements, which is most likely driven by environmental and lifestyle factors, might have a role in the pathogenesis of AMD. These trace elements may be incorporated as biomarkers into models for prediction of disease risk and progression. Additionally, population-based preventive strategies to decrease Cd exposure, especially by the cessation of smoking, could potentially reduce the burden of nAMD. Future studies are warranted to investigate whether supplementation of Cr would have a beneficial effect on nAMD.

## Highlights

• Analyses of 16 plasma trace element levels in neovascular AMD patients vs controls.
• Barium and cadmium are elevated in 236 cases compared to 236 age-matched controls.
• Chromium levels are reduced in the neovascular AMD patients compared to controls.
• Environmental and lifestyle factors affect trace-element levels in neovascular AMD.
• Trace elements provide a potential target for novel strategies for AMD prevention.

## 1 Introduction

Age-related macular degeneration (AMD) is a complex, multifactorial retinal disease and one of the leading causes of severe vision loss globally in those over 50 years of age (Jonas et al., 2017). As the demographic, environmental, genetic, and phenotypic underpinnings of AMD becomes better understood, a combination of innovative screening and assessment could enable the development of risk models and novel preventive strategies (Panattoni et al., 2011). The most consistently reported demographic, environmental and lifestyle risk factors for AMD include advanced age, smoking and obesity (Colijn et al., 2017; Velilla et al., 2013; Zhang et al., 2016). In addition, studies show mixed results regarding the involvement of the cardiovascular system in AMD, including hypertension, pulse pressure and circulating lipids (Hyman et al., 2000; Pennington and DeAngelis, 2016). Recently, 52 common and rare genetic variants at 34 loci have been associated with AMD, which particularly involve the complement system, lipid metabolism and extracellular matrix (Fritsche et al., 2016). The two most important phenotypic risk factors for disease progression in AMD are drusen and subretinal drusenoid deposits, which are extracellular deposits in the retina between the Bruch's membrane (BM) and retinal pigment epithelium (RPE), and RPE and photoreceptor layer, respectively (Chen et al., 2020). Patients with drusen and subretinal drusenoid deposits can eventually progress to late stage AMD, which can be distinguished as two subtypes: geographic atrophy (GA) and neovascular AMD (nAMD). While the development of GA involves a gradual degeneration of photoreceptors, RPE and the choriocapillaris, nAMD is characterized by a relatively rapid process of neovascularization from blood vessels in the choriod (i.e. under the macular region) into the retina. Although the triggers for angiogenesis in nAMD patients are not fully understood, inflammation and oxidative stress are likely involved.

Oxidative stress is an important contributor to the development of AMD. Oxidative stress in the eye can occur in many forms via a variety of stimuli. Local exposure of sunlight leads to a high predisposition for oxidative burden (Taylor et al., 1990). When combined with systemic exposure to oxidative stressors, incurred via lifestyle factors such as smoking, the relative burden of oxidative stress can rapidly become disproportionately high (Wang et al., 2009; Yuan et al., 2010). Oxidative stress generates reactive oxygen species (ROS) that damage the cell and matrix components in the RPE, BM, and choroid, and provides signals for inflammation and angiogenesis, leading to the development and progression of nAMD (Datta et al., 2017; Shaw et al., 2016).

Trace elements have been implicated as regulators of oxidative stress in other contexts (Stelling et al., 2019). Several studies have shown that metal ions such as cadmium (Cd), iron (Fe), and lead (Pb) can be detected in ocular tissues of human eyes (Dunaief, 2006; Erie et al., 2005; Langford-Smith et al., 2016). These trace elements are thought to be involved in the pathogenesis of nAMD by increasing chronic oxidative stress and decreasing antioxidant defense systems (World Health Organization, 1996). Cd has been found to be significantly higher in blood samples of smokers compared to non-smokers (Li et al., 2019), and is associated with a higher incidence of nAMD (Kim et al., 2016; Park et al., 2015). Furthermore, a secondary analysis of the Comparisons of AMD Treatments Trials showed that the use of iron supplements was associated with nAMD in a dose-response manner (Song et al., 2018). Other metal ions such as chromium (Cr), copper (Cu), magnesium (Mg), manganese (Mn), selenium (Se) and zinc (Zn) are known to be involved in cellular antioxidant defense mechanisms (Lee et al., 2019; Salmonowicz et al., 2014). In AMD patients, supplementation of Zn has shown to be beneficial in reducing the progression of the disease (Age-Related Eye Disease Study Research Group, 2001; van Asten et al., 2019). Moreover, elderly individuals have generally a higher risk to develop trace element deficiencies due to modified dietary habits, age related physiological changes, drug therapy, and chronic diseases (Ekmekcioglu, 2001). Together these findings suggest that there may be a role for altered levels of essential trace elements in modifying the progression toward nAMD.

Whilst direct measurement of trace element levels in the retina provides accurate and reliable data, tissue biopsies are invasive techniques, not easily obtained and not feasible for risk assessment in large populations. Conversely, systemic measurements are less invasive, quicker and more easily repeatable. Since blood is a source for trace elements entering the eye (Abokyi et al., 2020), it was hypothesized that analyzing plasma trace element levels in nAMD patients would be informative; i.e. especially as there is limited data available in the literature on plasma trace element levels in nAMD. Therefore, the main purpose of the current study was to identify novel systemic biomarkers for nAMD by investigating plasma metal ion and metalloid levels, comparing patients with nAMD and age-matched controls. Quantitative data were obtained for 13 metal ions (barium (Ba), calcium (Ca), Cd, cobalt (Co), Cr, Cu, Fe, Mg, Mn, molybdenum (Mo), Pb, vanadium (V) and Zn), 2 metalloids (arsenic (As) and antimony (Sb)) and one non-metal (Se) in plasma samples from 236 nAMD patients and 236 controls. Secondly, we explored the underlying mechanisms of our findings by investigating the association of the 16 plasma trace element levels with demographic, environmental and lifestyle factors, as well as with AMD-associated variants at 34 genomic loci.

## 2 Material and methods

### 2.1 Study participants

Study participants with available plasma samples were recruited from the European Genetic Database (EUGENDA), a multicenter database for clinical and molecular analysis of AMD from Nijmegen, the Netherlands, and Cologne, Germany. Written informed consent was obtained for all individuals participating in the study, which was performed according to the guidelines of the Declaration of Helsinki and with local ethics committee approval (Arnhem-Nijmegen Commission for Human Research). To prevent bias caused by the study site only individuals from the Nijmegen cohort were selected from the database. For every individual patient with neovascular AMD (nAMD), a control was manually identified from the database (with an age difference <0.5 years). According to this approach, we were able to include a total of 236 nAMD patients and 236 age-matched controls, who had entered the EUGENDA database between 2006 and 2015. All patients with nAMD had been treated in the past with injections of anti-angiogenic drugs. Retinal images were assessed by certified graders based on the protocol of the Cologne Image Reading Center and Laboratory (CIRCL) an AMD classification that was adopted specifically for EUGENDA based on different international staging systems (Bird et al., 1995; van Leeuwen et al., 2003). The diagnosis of nAMD was based on multimodal grading of images obtained via color fundus photographs (CFP) only (n = 24, [10%]), a combination of CFP and optical coherence tomography (OCT) (n = 105, [44%]), a combination of CFP and fluorescein angiography (FA), and/or indocyanine green angiography (ICGA) (n = 85, [36%]), or a combination of CFP, OCT and FA/ICGA (n = 22, [10%]). At the time of inclusion, patients with nAMD presented with choroidal neovascularization (CNV) within the Early Treatment Diabetic Retinopathy Study (ETDRS) grid secondary to AMD with or without signs of CNV activity (hemorrhage on CFP and/or leakage on FA and/or subretinal and/or intraretinal fluid on OCT). Controls included participants with no drusen, only small drusen (<63 μm), only pigmentary abnormalities, or <10 small drusen and pigment abnormalities, as defined by the CIRCL grading protocol (Heesterbeek et al., 2020).

### 2.2 Demographic, environmental and lifestyle factors

Information on demographic, environmental and lifestyle factors was obtained through detailed interviewer-assisted questionnaires. Variables included in this study were age in years (<65; 65–70; 70–75; 75+), sex (male; female), smoking (never; past; current), body mass index (BMI) calculated as weight in kilograms divided by height in meters squared (normal [<25]; overweight [25–30]; obese [>30]), having metal implants including hip or knee prosthesis (yes; no), using nutritional supplements (yes; no), using lipid lowering drugs (yes; no), using antidiabetic drugs (yes; no), using antihypertensive drugs (yes; no), using thrombocyte inhibitors (yes; no), using nonsteroidal anti-inflammatory drugs (NSAIDs; yes; no), using corticosteroids (yes; no), meat consumption in general (yes; no), fish consumption in general (yes; no), alcohol consumption in general (yes; no), daily fruit consumption (yes; no), and daily vegetable consumption (yes; no). In addition, during a physical examination, the degree of lens opacification was determined and quantified using the Lens Opacity Classification System (LOCS). Participants with a LOCS score 1–2 were categorized as having no cataract, while participants with a LOCS score 3–4 were categorized as having cataract.

### 2.3 Genetic variants

Genomic DNA was isolated from venous blood lymphocytes and genotyping was performed for AMD-associated variants at 34 loci using single molecule molecular inversion probes (smMIPs) and next generation sequencing as part of the Eyerisk Project (de Breuk et al., 2020). smMIPs are single stranded DNA molecules and contain sequences that are complementary to the target in the genome, which in this case allows the identification of alleles of the AMD-associated variants for every participant. Genetic variants analyzed in the current study included *CFH* (rs10922109 and rs570618), *COL4A3* (rs11884770), *ADAMTS9-AS2* (rs62247658), *COL8A1* (rs140647181), *CFI* (rs10033900), *C9* (rs62358361), *PRLR/SPEF2* (rs74767144), *C2/CFB/SKIV2L* (rs116503776), *VEGFA* (rs943080), *KMT2E/SRPK2* (rs1142), *PILRB/PILRA* (rs7803454), *TNFRSF10A* (rs79037040), *MIR6130/RORB* (rs10781182), *TRPM3* (rs71507014), *TGFBR1* (rs1626340), *ABCA1* (rs2740488), *ARHGAP21* (rs12357257), *ARMS2/HTRA1* (rs3750846), *RDH5/CD36* (rs3138141), *ACAD10* (rs61941272), *B3GALTL* (rs9564692), *RAD51B* (rs61985136), *LIPC* (rs2043085), *CETP* (rs5817082), *CTRB2/CTRB1* (rs55993634), *TMEM9*7/VTN (rs11080055), *NPLOC4/TSPAN10* (rs6565597), *C3* (rs2230199), *CNN2* (rs113772652), *APOE* (rs429358), *MMP9* (rs142450006), *C20orf85* (rs117420707), *SYN3/TIMP3* (rs5754227) and *SLC16A8* (rs8135665).

### 2.4 Trace element measurements

Plasma samples were collected using tubes containing anticoagulant and blood cells were removed by centrifugation; samples were processed and stored at -80 °C within 1 h of collection. Prior to trace element quantitation by inductively coupled plasma mass spectrometry (ICP-MS), plasma samples of cases and controls (150 μL), or serum controls from the National Institute of Standards and Technology (NIST) (150 μL; see below), were combined with a 1.3 mL solution of 1% (v/v) Ultrapure NORMATOM nitric acid (VWR; catalogue no. 83879.230), 0.5% (v/v) HPLC-grade butanol (Sigma; 34867), 0.01% (v/v) Triton X-100 (Sigma; X100-500ML) and 0.1% Agilent Internal Standard mixture (containing 10 μg/mL of bismuth (Bi), germanium (Ge), indium (In), scandium (Sc), terbium (Tb), yttrium (Y) and lithium-6 (Li) (in 5% (v/v) HNO_3_ (Agilent Technologies; 5183–4681)) in LC/MS grade water (Fisher Chemical; 10505904), mixed and then 50 μL of 2% (v/v) nitric acid was added, i.e. to give a final volume of 1.5 mL (1 in 10 dilution). This protocol was adapted from the method of Cesbron et al. (2013) and validated with reference to certified values for Cd, Co, Cu, Fe, Mn, nickel (Ni), Sb, Se, V and Zn in Standard Reference Material (SRM) 1598a from NIST, USA; NIST also supply reference values for aluminium (Al), Ca, Cr and Mo and an information value for As in this standard. A total of 18 trace elements were measured using ICP-MS: Al, As, Ba, Ca, Cd, Co, Cr, Cu, Fe, Mg, Mn, Mo, Ni, Pb, Sb, Se, V and Zn.

Trace element concentrations were measured using an Agilent 7700x ICP-MS spectrometer equipped with a MicroMist nebulizer (Glass Expansion, Melbourne, Australia) and a Scott double-pass spray chamber, using a multi-element method including all elements present in the calibration solution (Church et al., 2015; Xu et al., 2017). Calibration solutions were produced by appropriate dilutions of Environmental Calibration Standard (Agilent 5183–4688) for each batch of samples; a full range of standard concentrations were analyzed at the beginning of each batch with an intermediate concentration from this calibration series run approx. every 10 samples to ensure there is no instrument drift. Instrument and dilution blanks were also run periodically in each experiment and the detection limit for each element was determined by comparison of calibration samples and blanks. The instrument was tuned prior to each run according to manufacturer's recommendations using a tuning solution containing 10 μg/L Li, Y, cerium (Ce), thallium (Tl), and Co in 2% (v/v) HNO_3_ (Agilent; 5184–3566). Sample introduction was performed using an Agilent Integrated Autosampler (I-AS) with helium (He) as the collision gas. Ge was used as the internal standard for Mg, Al, Ca, V, Cr, Mn, Fe, Co, Ni, Cu, Zn, As and Se; In was used as the internal standard for Mo, Cd, Sb, Ba and Pb. Two collision cell gas modes were applied: all elements were analyzed in helium mode (5.0 mL/min He), except for Se that was analyzed in high-energy helium mode (10 mL/min He) and for Ge and In, which were analyzed in both modes; mode selection followed Agilent recommendations. Integration times were: 0.01 s for Fe; 0.1 s for Mg, Ca, Sb, Ba and Pb; 0.2 s for V and Co; 0.3 s for Mn, Cu, Zn and Mo; 0.5 s for Ni; 0.7 s for Cr; 1 s for Al and Cd; 1.5 s for As; and 3 s for Se. Sample trace element concentrations were automatically calculated by the software employed (Mass Hunter, Agilent). Data were exported to Microsoft Excel and corrected for dilution.

During the measurements, samples were divided into 10 batches consisting of 5 × 50, 3 × 52, 1 × 46 and 1 × 20 samples. Each batch included both cases and their age-matched controls. SRM 1598a from NIST, which is derived from a mixture of healthy bovine and porcine sera, was used as an internal validation control, with triplicate standards analyzed in each batch to provide evidence of accuracy of trace element measurements and reproducibility between batches (Supplementary Table 1); 5 blanks (prepared as for the samples and NIST standards but lacking plasma/serum) were also included. To minimize contamination high-grade solvents and metal-free plastics were used throughout. Contamination was measured by inclusion of ‘blank’ samples within each batch. Al and Ni measurements were excluded, since these metal ions exceeded a coefficient of variance of 40% in the SRM 1598a controls, which indicated inadequate accuracy and reproducibility between batches; this was attributed to levels below our limit of detection (for Ni; likely due to the use of Ni cones in the ICP-MS) or the presence of variable trace-level contamination (for Al). For the other trace elements, values that were measured below the limit of quantification (LOQ) in a particular batch were substituted by LOQ/2, since excluding these measurements would have introduced significant bias (Helsel, 1990). Substitution of these values was done in a batch-specific manner by using the LOQ of the particular batch. Measurements for 16 trace elements were included in the further analyses (Supplementary Table 1).

### 2.5 Statistical analysis

Statistical analyses were conducted using SPSS (IBM, version 25). Demographic differences between nAMD patients and controls were assessed using independent *t*-tests and chi-squared tests. Levels of normally distributed trace elements were compared between nAMD patients and controls using independent *t*-tests, whereas non-normally distributed trace elements were tested with a Kruskal-Wallis H test. In addition, to explore possible underlying mechanisms, the association of trace element levels with demographic, environmental, lifestyle and genetic factors was evaluated; again, independent t-tests for normally distributed trace elements and Kruskal Wallis H test for non-normally distributed trace elements were used. For all these statistical analyses, P values below 0.003 were considered statically significant, which corresponds to a Bonferroni correction for 16 covariates (Bender and Lange, 2001). The significant AMD-associated trace elements were thereafter analyzed in a multifactorial binary logistic regression model, adjusting for the relevant demographic, environmental and lifestyle factors.

To investigate the correlation between individual trace elements, a Spearman's rho rank correlation between the trace elements was calculated. Due to the strong correlations between trace elements, correction for multiple testing in this analysis was performed by applying the Benjamini-Hochberg procedure, a false discovery rate (FDR) correction (Benjamini and Hochberg, 1995). A significance threshold of P_FDR_-value <0.05 after FDR correction was applied to this analysis. Correlation coefficients between trace elements were compared between nAMD patients and controls using the “r. test” function of the “psych” package.

## 3 Results

### 3.1 Demographics

This study included 236 nAMD patients and 236 unaffected controls. Sex was not used as a selection criteria, nonetheless, by chance, the sex distribution was the same in the nAMD group and controls (56% female in both). The mean age of the nAMD patients and of the age-matched controls was 73.5 years. There were significantly more current smokers in the nAMD group compared to controls (22% vs 6%, P < 0.001). There was also a higher use of alcohol (40% vs 31%, P = 0.043), supplements (36% vs 10%, P < 0.001), antidiabetic drugs (9% vs 4%, P = 0.016) and antihypertensive drugs (47% vs 37%, P = 0.032) among patients with nAMD compared to controls (Table 1). In addition, nAMD patients were more frequently pseudophakic (i.e. having had their lens removed during cataract surgery) compared to controls (30% vs 18%, P = 0.012), but phakic nAMD patients (i.e. with natural lenses) did not have a greater prevalence of cataract compared to phakic controls (32% vs 37%, P = 0.382). Other demographic parameters (including sex, BMI and presence of metal implants) did not differ significantly between cases and controls.

**Table 1:** Demographic table.

	Controls (n = 236)	nAMD patients (n = 236)	P-value
Age in years (mean (SD))	73.48 (6.70)	73.49 (6.67)	0.993

Sex, Female (n, (%))	132 (56%)	132 (56%)	1.0

BMI (mean (SD))	26.0 (3.8)	26.4 (3.7)	0.175

Smoking status (n, (%))			<0.001
Never	88 (37%)	66 (28%)	
Past	133 (57%)	119 (50%)	
Current	15 (6%)	51 (22%)	

Daily vegetables consumption (n, (%))			0.889
Yes	206 (87%)	207 (88%)	
No	30 (12%)	29 (12%)	

Daily fruit consumption (n, (%))			0.117
Yes	192 (81%)	178 (75%)	
No	44 (19%)	58 (25%)	

Meat consumption (n, (%))			0.765
Yes	212 (90%)	210 (89%)	
No	24 (10%)	26 (11%)	

Fish consumption (n, (%))			0.450
Yes	183 (78%)	176 (75%)	
No	53 (22%)	60 (25%)	

Alcohol consumption (n, (%))			0.043
Yes	73 (31%)	94 (40%)	
No	163 (69%)	142 (60%)	

Supplement intake (n, (%))			<0.001
Yes	24 (10%)	86 (36%)	
No	212 (90%)	150 (64%)	

Lipid lowering drugs (n, (%))			0.246
Yes	55 (23%)	66 (28%)	
No	181 (77%)	170 (72%)	

Antidiabetic drugs (n, (%))			0.016
Yes	9 (4%)	22 (9%)	
No	227 (96%)	214 (91%)	

Antihypertensive drugs (n, (%))			0.032
Yes	87 (37%)	110 (47%)	
No	149 (63%)	126 (53%)	

Thrombocyte inhibitors (n, (%))			0.088
Yes	41 (17%)	56 (24%)	
No	195 (83%)	180 (76%)	

NSAIDs (n, (%))			0.823
Yes	10 (4%)	11 (5%)	
No	226 (96%)	225 (95%)	

Corticosteroids (n, (%))			0.356
Yes	20 (8%)	27 (11%)	
No	216 (92%)	209 (89%)	

Metal implants (n, (%))			0.204
Yes	33 (14%)	24 (10%)	
No	203 (86%)	212 (90%)	

Lens status (n, (%))			0.012
Pseudophakic	32 (18%)	48 (30%)	
Phakic	142 (82%)	111 (70%)	

If Phakic, having cataract			0.382
No (LOCS 1–2)	97 (68%)	70 (63%)	
Yes (LOCS 3–4)	45 (32%)	41 (37%)	

Analyses are based on independent t-tests and chi-square tests. Abbreviations: BMI, Body Mass Index; LOCS, lens opacity classification system; nAMD, neovascular age-related macular degeneration; NSAIDs, nonsteroidal anti-inflammatory drugs.

### 3.2 Quality control

In our study, we obtained quality controlled ICP-MS data for 16 trace elements (Supplementary Table 1). For Ca, Cd, Cu, Fe, Sb, Se, V and Zn the mean values determined for these trace elements in the NIST controls were within 15% of supplied values; for As, Co, Cr, Mn and Mo they were within 22–35%. Mean values for Ba and Mg in the nAMD case-control samples, for which reference values were not available, were in good agreement with published data, whereas the levels of Pb were somewhat lower than some reported previously (Bergdahl et al., 2006; Cesbron et al., 2013; Jahnen-Dechent and Ketteler, 2012). Three of the 16 trace elements included in the analysis had values outside the LOQ [Cd (165 samples, 35%), Pb (213 samples, 45%) and V (54 samples, 12%)]. For V, the distribution of values below the LOQ was essentially identical between nAMD patients (6%) and controls (6%). However, for Cd and Pb the values below the LOQ were more frequently found in controls; 15% in nAMD vs 20% in controls for Cd (P = 0.015), and 20% in nAMD vs 25% in controls for Pb (although not significant; P = 0.065). All trace elements, except As, Cd and Pb, showed a normal distribution in patients and controls. Therefore, parametric tests were used for the analysis of all trace elements, except for As, Cd and Pb, for which non-parametric tests were used instead.

### 3.3 Association between trace elements and neovascular AMD

Patients with nAMD had higher plasma Ba and Cd levels, and lower plasma Cr levels compared to controls (Table 2). Mean levels for Ba were 1.35 μg/L (standard deviation [SD] 0.71) in nAMD patients and 1.15 μg/L (SD 0.63) in controls (P = 0.001) and mean levels of Cr were 0.37 μg/L (SD 0.22) in nAMD patients and 0.46 μg/L (SD 0.34) in controls (P = 0.001). Median levels for Cd, which were not normally distributed, were 0.016 μg/L (interquartile range [IQR] 0.001–0.026) in nAMD patients and 0.012 μg/L (IQR 0.001–0.022) in controls (P = 0.002).

**Table 2:**
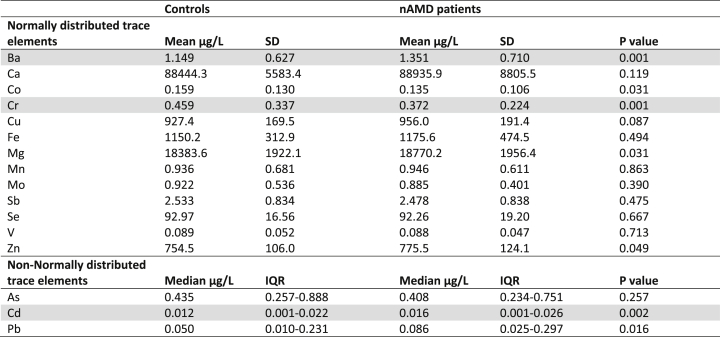
Results of plasma trace elements in 236 nAMD patients compared to 236 controls.

Analyses are based on independent t-tests for normally distributed trace elements, and Kruskal-Wallis H tests for non-normally distributed trace elements. Grey bars denote trace elements for which there was a significant difference between nAMD patients and controls after applying Bonferroni correction for 16 covariates (the bonferoni adjusted threshold for statistical significance was defined as P < 0.003). Abbreviations: As, arsenic; Ba, barium; Ca, calcium; Cd, cadmium; Co, cobalt; Cr, chromium; Cu, copper; Fe, iron; IQR, interquartile range; Mg, magnesium; Mn, manganese; Mo, molybdenum; nAMD, neovascular age-related macular degeneration; Pb, lead; Sb, antimony; SD, standard deviation; Se, selenium; V, vanadium; Zn, zinc.

### 3.4 Correlation among trace elements

**Fig. 1:**
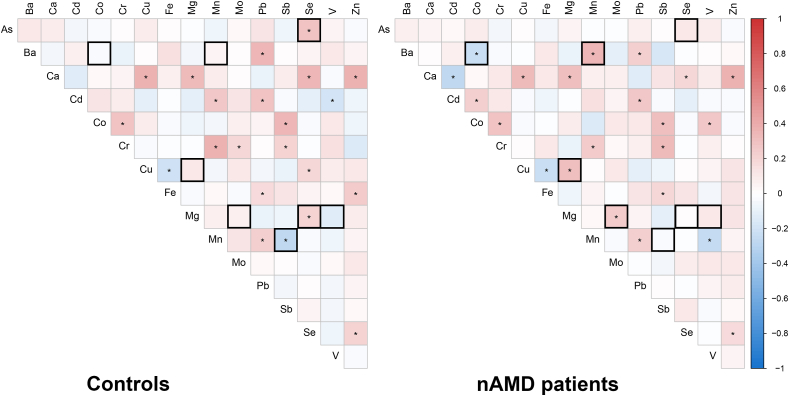
Spearman's correlation between plasma trace elements in nAMD patients and controls. Left-hand side: Correlations are depicted as observed in the controls. Right-hand side: Correlations in nAMD patients. Colors indicate positive (red) and negative (blue) correlations between the different trace elements, and significant correlations (P_FDR_-value <0.05) after FDR correction are indicated with an asterix (*). Black bordered squares are those correlations that were significantly different between nAMD patients and controls (Table 3). Supplementary Table 2 provides all plasma trace element correlations in nAMD patients and controls in more detail. Abbreviations: As, arsenic; Ba, barium; Ca, calcium; Cd, cadmium; Co, cobalt; Cr, chromium; Cu, copper; Fe, iron; Mg, magnesium; Mn, manganese; Mo, molybdenum; nAMD, neovascular age-related macular degeneration; Pb, lead; Sb, antimony; Se, selenium; V, vanadium; Zn, zinc. (For interpretation of the references to color in this figure legend, the reader is referred to the Web version of this article.)

Next, we analyzed the correlation among plasma trace elements. After applying FDR correction, Spearman's rho correlations showed several trace elements that were significantly correlated in both nAMD patients and controls (Fig. 1 and Supplementary Table 2). Comparison of the Spearman's correlation coefficients between nAMD patients and controls identified a difference in correlations for 8 trace elements. For example, there is a negative correlation between Ba and Co levels and a positive correlation between Ba and Mn levels in the nAMD cohort, which is not seen in controls (Table 3).

**Table 3:** Significant differences in the correlations for plasma trace elements between nAMD patients and controls.

	Controls		nAMD patients		Correlation difference	
Correlation	Spearman's Rho	P_FDR_-value	Spearman's Rho	P_FDR_-value	Delta Rho	P_FDR_-value
As – Se	0.295	<0.001	0.115	0.275	0.180	0.042
Ba – Co	-0.028	0.841	-0.224	0.004	0.196	0.031
Ba – Mn	0.040	0.752	0.358	<0.001	0.318	<0.001
Cu – Mg	0.113	0.294	0.315	<0.001	0.202	0.022
Mg – Mo	0.065	0.591	0.246	0.001	0.181	0.045
Mg – Se	0.206	0.010	0.004	0.979	0.202	0.027
Mg – V	-0.136	0.164	0.114	0.282	0.250	0.007
Mn – Sb	-0.263	<0.001	-0.026	0.865	0.237	0.009

Analysis is based on a spearman's rho rank correlation between the different trace elements and adjusted for multiple testing using FDR correction. The threshold of statistical significance was defined as P_FDR_-value <0.05 after FDR correction. Significant correlation coefficients of nAMD patients and controls were compared using the “r. test” function of the “psych” package. Supplementary Table 2 provides all plasma trace element correlations. Abbreviations: As, arsenic; Ba, barium; Co, cobalt; Cu, copper; Mg, magnesium; Mn, manganese; Mo, molybdenum; nAMD, neovascular age-related macular degeneration; Sb, antimony; Se, selenium; V, vanadium.

### 3.5 Association between trace elements and demographic, environmental and lifestyle factors

Then, we investigated the association of the 16 trace elements with several demographic, environmental and lifestyle factors (Table 4). P values below 0.003 were considered statically significant, which corresponds to a Bonferroni correction for 16 covariates. Cd levels were significantly associated with the smoking status of the participants (never smoked 0.011 μg/L, smoked in the past 0.014 μg/L and current smoking 0.021 μg/L, P < 0.001). There was a trend for higher Ba levels in patients using antihypertensive drugs compared to patients not using such drugs (1.355 μg/L vs 1.173 μg/L, P = 0.005), however, this trend did not reach the Bonferroni significance threshold. Cr was not significantly associated with any of the investigated demographic, environmental and lifestyle factors (P > 0.003).

**Table 4:**
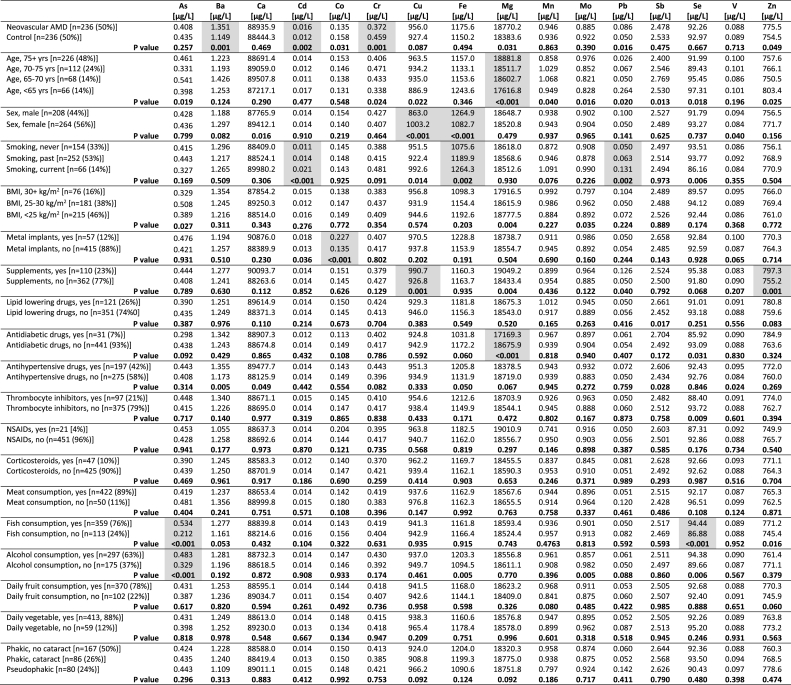
Association between plasma trace element levels and demographic, environmental and lifestyle factors.

Analyses are based on ANOVA t-tests for normally distributed trace elements, and Kruskal-Wallis H tests for non-normally distributed trace elements. Grey boxes denote trace elements for which there was a significant difference in plasma trace element levels between demographic categories after applying Bonferroni correction for 16 covariates (the Bonferroni adjusted threshold for statistical significance was defined as P < 0.003). Abbreviations: ANOVA, Analysis of variance; As, arsenic; Ba, barium; BMI, Body Mass Index; Ca, calcium; Cd, cadmium; Co, cobalt; Cr, chromium; Cu, copper; Fe, iron; Mg, magnesium; Mn, manganese; Mo, molybdenum; nAMD, neovascular age-related macular degeneration; Pb, lead; Sb, antimony; Se, selenium; V, vanadium; Zn, zinc.

For the non-AMD associated trace elements, higher age was associated with higher Mg levels, female sex was associated with higher Cu levels and lower Fe levels, smoking was associated with higher Fe levels and higher Pb levels, having metal implants was associated with higher Co levels, using supplements was associated with higher Cu levels and higher Zn levels, using antidiabetic drugs was associated with lower Mg levels, fish consumption was associated with higher As levels and higher Se levels, and alcohol consumption was associated with higher Se levels. As shown in Table 4, all of these trace elements were significantly associated with these demographic, environmental or lifestyle factors (P < 0.003), but they were not significantly associated with nAMD in this study (Table 2). None of the trace elements were significantly associated with having cataract in combined nAMD patients and controls (Table 4) or when just controls were analyzed (Supplementary Table 3).

### 3.6 Association between trace elements and genetic variants

Next, we analyzed the association of the 16 trace elements with AMD-associated variants, in which we also considered a P value below 0.003 as statistically significant. As can be seen in Supplementary Table 4, none of the AMD-associated variants at the 34 genomic loci were significantly associated with any of the trace elements (P > 0.003).

### 3.7 Risk model for neovascular AMD based on trace elements

Finally, multivariate logistic regression was used for estimating the odds of having nAMD based on the plasma values of Ba, Cd and Cr, adjusting for all demographic, environmental and lifestyle confounders including: age, sex, smoking status, BMI, having metal implants, using supplements, lipid lowering drugs, antidiabetic drugs, thrombocyte inhibitors, NSAIDs and corticosteroids, and consuming meat, fish, alcohol, fruit and vegetables. As can be seen in Table 5, all three trace elements remained significantly associated with nAMD after adjustment for the confounders (Ba: OR 1.55 per μg/L, Cd: OR 1.02 per ng/L, and Cr: OR 0.09 per μg/L).

**Table 5:** Plasma trace elements associated with nAMD.

	Estimate (β)	Odds Ratio	P value
Barium (μg/L)	0.435	1.545	0.019
Cadmium (ng/L)	0.022	1.022	0.006
Chromium (μg/L)	-2.466	0.085	0.001

Binary logistic regression analysis, adjusted for following confounders: age (continuous), sex (male/female), smoking status (never/past/current), BMI (continuous), having metal implants (yes/no), using supplements (yes/no), using lipid lowering drugs (yes/no), using antidiabetic drugs (yes/no), using thrombocyte inhibitors (yes/no), using NSAIDs (yes/no), using corticosteroids (yes/no), meat consumption in general (yes/no), fish consumption in general (yes/no), alcohol consumption in general (yes/no), daily fruit consumption in general (yes/no), daily vegetable consumption in general (yes/no), barium (μg/L), cadmium (ng/L), and chromium (μg/L).

The odds ratio for continuous variables should be interpreted as odds per point of increase of the variable. For example, when the barium level increases with 0.50 μg/L, the risk of having nAMD is 1.545^0.50^ = 1.243 or 24.3% higher.

Abbreviations: BMI, Body Mass Index; nAMD, neovascular age-related macular degeneration; NSAIDs, nonsteroidal anti-inflammatory drugs.

## 4 Discussion

In order to obtain a comprehensive overview of circulating trace elements in nAMD, we analyzed 16 plasma metal ions, metalloids and a non metal in 236 patients with nAMD and 236 age-matched controls using mass spectrometry. We detected elevated plasma levels of Ba and Cd and reduced plasma levels of Cr in nAMD patients compared to controls. Additional analysis showed that Cd levels were predominantly associated with the smoking status of participants, and Ba levels showed a trend towards association with the usage of antihypertensive drugs. Cr levels was not associated with any of the demographic, environmental and lifestyle factors. The AMD-associated genetic variants were not associated with any of the plasma trace elements.

A limited number of studies have been performed to date on circulating trace element levels in AMD patients, and these studies focused on either single measurements or at most five trace elements simultaneously (Aranaz et al., 2020; Cardinault et al., 2005; Damar Gungor et al., 2018; Kim et al., 2014, 2016; Mayer et al., 1998; Park et al., 2015; Tsang et al., 1992; Wu et al., 2014; Wysokinski et al., 2013). In line with our results, most of these studies found higher systemic Cd levels in AMD patients, especially within the smoking population (Kim et al., 2014, 2016; Wu et al., 2014). These results are also consistent with histology studies, which showed Cd accumulation in the neurosensory retina and RPE of eyes affected by AMD and in those who smoked (Wills et al., 2008, 2009). Cd is thought to gain access to the RPE via transport from the choroid by carrier proteins or metal transport mechanisms, such as zinc transporters ZIP4 and ZIP8 at the basal surface of the RPE that have a high affinity for Cd (Girijashanker et al., 2008; He et al., 2006). Within RPE cells, Cd is also able to interfere with the metabolism of Zn by competing for its binding to metallothioneins, which display a much greater affinity for Cd than for Zn (Gonzalez-Iglesias et al., 2014). Zn made bioavailable in this way could also potentially affect complement activity; e.g. by binding to complement factor H and C3, thereby inhibiting factor H's cofactor activity and precipitating protein complexes that might contribute to the formation of drusen and drusenoid deposits (Day and Sim, 1986; Lengyel et al., 2007; Nan et al., 2013). Additionally, because antioxidant and visual cycle enzymes in the retina require Zn, the interference of Cd could have widespread deleterious effects on retinal functions (Rink, 2011; Ugarte and Osborne, 2001). Furthermore, Cd can enhance production of ROS which results in DNA and protein damage, inflammation, apoptosis and angiogenesis (Cuypers et al., 2010). Population-based preventive strategies to decrease Cd exposure, especially by the cessation of smoking, could reduce the burden of nAMD.

To our knowledge, no studies have been published that have reported higher plasma levels of Ba in nAMD patients as observed in the current work. Some epidemiological investigations show a positive correlation between Ba exposure and hypertension (Nigra et al., 2016), which is in line with the trend in our study in which higher Ba levels were found in participants using antihypertensive drugs. It has been reported that Ba can inhibit potassium (K) channels of the sodium-potassium (Na/K) pump in smooth muscle cells of arteries and arterioles, leading to reduced blood flow (Tykocki et al., 2017). This suggests that higher circulating Ba levels might be associated with nAMD by a reduced blood flow in the choroid, where Ba is reported to be present (Sowden and Pirie, 1958). Furthermore, electrophysiology studies in human donor eyes also show that Ba is able to inhibit the Na/K pump at the apical and basolateral membranes of the RPE, thereby reducing the transport of molecules across RPE cell membranes (Quinn and Miller, 1992). The inverse relationship between Ba and Co levels seen in nAMD patients could also be of relevance, given that there is evidence that Co can induce expression of vascular endothelial growth factor (VEGF) in RPE cells (Zhang et al., 2015). However, the direct role of Ba in the etiology of nAMD is currently unknown, and further research is needed to determine the influence of elevated Ba levels on the function of RPE and choroid.

Despite our observation of lower levels of Cr in nAMD patients, we were not able to identify underlying associations with demographic, environmental, lifestyle or genetic factors. Studies in Cr-deficient rats showed more phagocytized lamellar structures in the RPE, providing evidence that Cr has a beneficial effect on the physiology of the RPE (Ueda et al., 2002). Furthermore, a study in hens showed that supplementation of Cr can increase serum vitamin C and E levels, and decrease malondialdehyde (MDA) levels( Onderci et al., 2003). MDA, which is a byproduct of polyunsaturated fatty acid peroxidation, has been implicated in RPE dysfunction and in driving angiogenesis through the stimulation of VEGF production by RPE cells (Weismann et al., 2011; Ye et al., 2016). Thus, it is conceivable that a reduction in circulating Cr levels could contribute to nAMD pathology, through a loss of its protective function and induction of pro-angiogenic pathways. Several studies report associations between AMD and diabetes mellitus, and hypothesize a common disease pathway of oxidative stress caused by hyperglycemia and dyslipidemia leading to macular pathology (Chen et al., 2014). In type 2 diabetes mellitus, Cr supplementation has a significant favorable effect on fasting glucose levels and total cholesterol levels. Whether supplementation of Cr is also beneficial for altering the disease progression towards nAMD is not known. In this regard, it would be informative to explore whether Cr levels are also reduced at a local level in the RPE.

In AMD, supplementation of Zn has shown to be beneficial in reducing the progression of the disease (Age-Related Eye Disease Study Research Group, 2001; Chew et al., 2015; Seddon et al., 2016). In our study, we detected higher plasma Zn levels in participants using supplements, but we did not detect significant differences in Zn levels between nAMD patients and controls. This might be due to the fact that only 36% of the nAMD patients reported supplement usage. The empirical lower limit of normal plasma Zn has been reported to be 750 μg/L, which is very similar to the mean plasma Zn level in controls from our study (Table 2) (Yokoi et al., 2003). However, the plasma Zn level is probably not a sensitive indicator of the Zn status of an individual since this only represents ~0.1% of the whole-body Zn concentration (Mills, 1988). For instance, in drusen, the local Zn levels are much higher, with a previous study reporting an average concentration of 500 ppm (equal to 500,000 μg/L) (Lengyel et al., 2007). Thus, measuring plasma Zn levels might not provide an accurate reflection of the local Zn levels in the macula of AMD patients. In addition, we analyzed equal volumes of plasma and calculated the amount of trace element per unit volume, which is in line with standard clinical biochemical practice. However, altered amounts of metal-binding proteins could influence the plasma levels of certain trace elements (including Zn). Therefore, future studies may also take into account the total level of plasma proteins or specific metal carriers, which may provide further mechanistic insights.

Here, by simultaneously measuring 16 trace elements by ICP-MS in a group of nAMD patients and controls, a comprehensive overview of systemic trace element levels was obtained. This approach allowed us to identify elevated levels of Ba and Cd and decreased levels of Cr in nAMD patients compared to controls. Several steps were taken in order to prevent confounding factors that are known to influence trace element levels. Controls used in this study were age-matched with the nAMD patients. Also, demographic, environmental, lifestyle and genetic factors were taken into account to investigate underlying associations with plasma trace element levels. However, other environmental risk factors such as the living environment of participants (rural versus urban) or performing industrial or nonindustrial work, might also influence plasma trace element levels but were not taken into account.

Even though this is one of the largest studies on trace element measurements in nAMD patients to date, our study should be considered as exploratory, i.e. to provide leads for follow-up research. Future studies should focus on replication of the findings of the current study in an independent nAMD cohort; for example, a recent method that uses a smaller sample volume (~30 μL) may prove useful to investigate the levels of particular metal ions (e.g. Cu, Fe and Zn)(Abduljabbar et al., 2019). Secondly, since we measured trace elements in plasma, this might not reflect the local levels in the eye. In this regard, donor eyes from patients with AMD were reported to have significantly higher levels of As, Ca, Cr, Pb, Ni and Se in choroid-RPE compared to non-AMD eyes (Aberami et al., 2019). Also, patients with AMD were found to have higher levels of Fe and lower levels of Zn within photoreceptors and RPE, compared to controls (Dunaief, 2006; Galin et al., 1962; Hahn et al., 2003). Based on our results, further investigation of Ba and Cr in donor nAMD eyes may be of relevance. In addition, despite AMD involving mainly disease processes in the macula, also drusen and subretinal drusenoid deposits can be identified in the periphery. Moreover, RPE cells show high topographical heterogeneity between the macula and peripheral retina (Burke and Hjelmeland, 2005). Therefore, future studies could be designed to investigate topographical differences in trace element levels in different regions of the retina. Finally, as only nAMD patients were included in this study, measurement of trace elements in patients with intermediate stage AMD, geographic atrophy, or subtypes of neovascularization could provide new insights into the pathophysiology of AMD and disease progression. Studies investigating phenotypic characteristics show that intraretinal neovascularization is strongly associated with subretinal drusenoid deposits, whereas subretinal and sub-RPE neovascularization is associated with drusen (Rabiolo et al., 2017). Therefore, it would be interesting to explore plasma trace element levels between these nAMD subtypes to better understand differences in their pathophysiology. Unfortunately, we were unable to carry out such an analysis here since OCT was only available for our EUGENDA studies from 2010; therefore we were unable to grade the subtype of neovascularization for half of the nAMD patients.

## 5 Conclusion

In conclusion, in this study we detected elevated levels of barium and cadmium and reduced levels of chromium in the plasma of nAMD patients. This study suggests that an imbalance in plasma trace elements might have a role in the pathogenesis of nAMD. These trace elements may be incorporated as biomarkers into models for prediction of disease risk and progression. Because plasma trace element levels are modifiable risk factors, this could lead to preventive interventions. Additionally, population-based preventive strategies to decrease Cd exposure, especially by the cessation of smoking, could potentially reduce the burden of nAMD. Future studies are warranted to investigate whether supplementation of Cr would have a beneficial effect on nAMD.

## Financial support

This work was co-funded by the 10.13039/501100000265Medical Research Council and the 10.13039/501100000266Engineering and Physical Sciences Research Council grant MR/N00583X/1 “Manchester Molecular Pathology Innovation Center (MMPathIC): bridging the gap between biomarker discovery and health and wealth” to AJD.

Dutch Research Council (016.Vici.170.024 to AIdH).

Oogfonds, Landelijke Stichting voor Blinden en Slechtzienden, Macula Fonds, Vereniging Bartiméus Sonneheerdt (Uitzicht, 2016–02 and 2016–26 to AIdH, CBH).

This project has also received funding from the European Union's Horizon 2020 research and innovation program under grant agreement No. 634479 (EYE-RISK).

## Declaration of competing interest

The authors have no proprietary or commercial interest in any materials discussed in this article.
