# Supplementary material for: Association of plasma trace element levels with neovascular age-related macular degeneration
Source: Exp Eye Res. 2020 Dec;201:108324. doi: 10.1016/j.exer.2020.108324 (PMC7773981; doi:10.1016/j.exer.2020.108324)
Supplement: Multimedia component 1 [file mmc1.docx]

**Association of Plasma Trace Element Levels with Neovascular Age-Related Macular Degeneration**

Thomas J. Heesterbeek, Mansour Rouhi-Parkouhi, Stephanie J. Church, Yara T. Lechanteur, Laura Lorés-Motta, Nikolaos Kouvatsos, Simon J. Clark, Paul N. Bishop, Carel B. Hoyng, Anneke I. den Hollander, Richard D. Unwin, Anthony J. Day

**Supplementary Tables**

**Supplementary Table 1. Quality control analysis of the NIST samples and overview of the LOQs**

| **Trace elements** | **NIST ± SD^a^**  **(µg/L)** | **EXP ± SD^b^**  **(µg/L)** | **CV^c^**  **(%)** | **LOQ^d^**  **(µg/L)**  **mean (min-max)** | **Excluded/**  **Included measurements** |
| --- | --- | --- | --- | --- | --- |
| Al | 2.3 ± 0.6 | 4.528 ± 2.674 | 59.1 | 0.136 (0.100-0.200) | Excluded |
| As | 0.3 ± NDS | 0.461 ± 0.121 | 26.3 | 0.005 (0.002-0.010) | Included |
| Ba | NDS | 6.081 ± 0.431 | 7.1 | 0.030 (0.010-0.050) | Included |
| Ca | 96000 ± 7000 | 92710 ± 6021.0 | 6.5 | 17.27 (10.00-20.00) | Included |
| Cd | 0.048 ± 0.004 | 0.048 ± 0.016 | 32.5 | 0.001 (0.001-0.001) | Included |
| Co | 1.24 ± 0.07 | 0.942 ± 0.064 | 6.8 | 0.002 (0.001-0.002) | Included |
| Cr | 0.33 ± 0.08 | 0.487 ± 0.141 | 29.0 | 0.011 (0.010-0.020) | Included |
| Cu | 1580 ± 90 | 1391.9 ± 95.4 | 6.9 | 0.264 (0.100-0.500) | Included |
| Fe | 1680 ± 60 | 1567.4 ± 93.6 | 6.0 | 1.33 (0.10-5.00) | Included |
| Mg | NDS | 20130.0 ± 1346.1 | 6.7 | 1.29 (0.20-5.00) | Included |
| Mn | 1.78 ± 0.33 | 2.28 ± 0.713 | 31.3 | 0.015 (0.010-0.050) | Included |
| Mo | 5.5 ± 1.0 | 3.878 ± 0.231 | 6.0 | 0.004 (0.001-0.010) | Included |
| Ni | 0.94 ± 0.18 | 0.866 ± 0.383 | 44.3 | 0.026 (0.010-0.100) | Excluded |
| Pb | NDS | 1.198 ± 0.350 | 29.2 | 0.025 (0.005-0.100) | Included |
| Sb | 1.00 ± 0.15 | 0.853 ± 0.086 | 10.1 | 0.014 (0.005-0.050) | Included |
| Se | 134.4 ± 5.8 | 150.9 ± 12.6 | 8.4 | 0.038 (0.010-0.100) | Included |
| V | 1.88 ± 0.11 | 2.069 ± 0.110 | 5.3 | 0.004 (0.001-0.020) | Included |
| Zn | 880 ± 24 | 791.3 ± 51.5 | 6.5 | 0.836 (0.200-2.000) | Included |

^a^Average values and range supplied by NIST for SRM 1598a. ^b^Experimental mean values determined here for 30 NIST samples (3 in each of 10 batches). ^c^Coefficient of variance of the experimental mean values for the 30 NIST samples. ^d^LOQ values (on instrument) across 10 batches. Abbreviations: Al, aluminum; As, arsenic; Ba, barium; Ca, calcium; Cd, cadmium; Co, cobalt; Cr, chromium; Cu, copper; CV, coefficient of variance; Fe, iron; LOQ: limit of quantification; Mg, magnesium; Mn, manganese; Mo, molybdenum; nAMD, neovascular age-related macular degeneration; NDS, no data supplied; Ni, nickel; NIST, National Institute of Standards and Technology; Pb, lead; Sb, antimony; SD, standard deviation; Se, selenium; V, vanadium; Zn, zinc.

**Supplementary Table 2.** Correlations of plasma trace elements in nAMD patients and controls

|  | **Controls** | | **nAMD patients** | | **Correlation difference** | |
| --- | --- | --- | --- | --- | --- | --- |
| **Correlation** | **Spearman’s**  **Rho** | **P_FDR_-**  **value** | **Spearman’s**  **Rho** | **P_FDR_-**  **value** | **Delta**  **Rho** | **P_FDR_-**  **value** |
| As – Ba | 0.109 | 0.299 | 0.074 | 0.503 | 0.036 | 0.699 |
| As – Ca | 0.083 | 0.468 | 0.048 | 0.754 | 0.035 | 0.705 |
| As – Cd | -0.056 | 0.658 | 0.030 | 0.838 | 0.086 | 0.354 |
| As – Co | -0.026 | 0.836 | 0.095 | 0.339 | 0.121 | 0.191 |
| As – Cr | 0.014 | 0.918 | -0.053 | 0.706 | 0.067 | 0.468 |
| As – Cu | 0.094 | 0.410 | 0.010 | 0.992 | 0.084 | 0.362 |
| As – Fe | 0.007 | 0.940 | -0.043 | 0.784 | 0.050 | 0.587 |
| As – Mg | -0.012 | 0.923 | 0.044 | 0.787 | 0.056 | 0.542 |
| As – Mn | -0.078 | 0.503 | -0.018 | 0.925 | 0.059 | 0.521 |
| As – Mo | 0.022 | 0.859 | -0.010 | 0.977 | 0.032 | 0.729 |
| As – Pb | 0.132 | 0.182 | 0.072 | 0.527 | 0.060 | 0.511 |
| As – Sb | -0.062 | 0.629 | 0.005 | 0.981 | 0.067 | 0.470 |
| As – Se | 0.295 | <0.001 | 0.115 | 0.275 | 0.180 | 0.042 |
| As – V | 0.046 | 0.715 | 0.094 | 0.343 | 0.048 | 0.600 |
| As – Zn | -0.023 | 0.852 | -0.032 | 0.829 | 0.009 | 0.927 |
| Ba – As | 0.109 | 0.299 | 0.074 | 0.503 | 0.036 | 0.699 |
| Ba – Ca | -0.047 | 0.706 | 0.001 | 0.982 | 0.049 | 0.599 |
| Ba – Cd | 0.062 | 0.621 | 0.078 | 0.479 | 0.016 | 0.866 |
| Ba – Co | -0.028 | 0.841 | -0.224 | 0.004 | 0.196 | 0.031 |
| Ba – Cr | -0.083 | 0.483 | -0.037 | 0.834 | 0.046 | 0.618 |
| Ba – Cu | 0.020 | 0.858 | -0.016 | 0.937 | 0.035 | 0.704 |
| Ba – Fe | 0.156 | 0.089 | 0.112 | 0.277 | 0.044 | 0.631 |
| Ba – Mg | -0.052 | 0.654 | -0.100 | 0.306 | 0.048 | 0.605 |
| Ba – Mn | 0.040 | 0.752 | 0.358 | <0.001 | 0.318 | <0.001 |
| Ba – Mo | 0.013 | 0.920 | -0.101 | 0.306 | 0.114 | 0.217 |
| Ba – Pb | 0.345 | <0.001 | 0.224 | 0.004 | 0.120 | 0.157 |
| Ba – Sb | 0.019 | 0.863 | -0.162 | 0.069 | 0.180 | 0.050 |
| Ba – Se | 0.045 | 0.709 | -0.002 | 0.984 | 0.047 | 0.611 |
| Ba – V | 0.111 | 0.301 | 0.010 | 0.962 | 0.101 | 0.275 |
| Ba – Zn | 0.043 | 0.728 | 0.033 | 0.833 | 0.010 | 0.910 |
| Ca – As | 0.083 | 0.468 | 0.048 | 0.754 | 0.035 | 0.705 |
| Ca – Ba | -0.047 | 0.706 | 0.001 | 0.982 | 0.049 | 0.599 |
| Ca – Cd | -0.136 | 0.170 | -0.261 | 0.001 | 0.125 | 0.160 |
| Ca – Co | 0.020 | 0.864 | 0.027 | 0.861 | 0.007 | 0.943 |
| Ca – Cr | 0.057 | 0.661 | 0.112 | 0.284 | 0.055 | 0.548 |
| Ca – Cu | 0.372 | <0.001 | 0.339 | <0.001 | 0.033 | 0.685 |
| Ca – Fe | 0.075 | 0.521 | 0.112 | 0.271 | 0.037 | 0.691 |
| Ca – Mg | 0.323 | <0.001 | 0.308 | <0.001 | 0.015 | 0.859 |
| Ca – Mn | 0.089 | 0.463 | 0.067 | 0.565 | 0.022 | 0.815 |
| Ca – Mo | 0.003 | 0.964 | 0.076 | 0.485 | 0.073 | 0.427 |
| Ca – Pb | -0.087 | 0.453 | -0.126 | 0.212 | 0.038 | 0.675 |
| Ca – Sb | 0.099 | 0.358 | 0.129 | 0.197 | 0.030 | 0.742 |
| Ca – Se | 0.349 | <0.001 | 0.191 | 0.020 | 0.158 | 0.065 |
| Ca – V | -0.035 | 0.801 | 0.087 | 0.403 | 0.122 | 0.187 |
| Ca – Zn | 0.366 | <0.001 | 0.366 | <0.001 | 0.001 | 0.998 |
| Cd – As | -0.056 | 0.658 | 0.030 | 0.838 | 0.086 | 0.354 |
| Cd – Ba | 0.062 | 0.621 | 0.078 | 0.479 | 0.016 | 0.866 |
| Cd – Ca | -0.136 | 0.170 | -0.261 | 0.001 | 0.125 | 0.160 |
| Cd – Co | 0.133 | 0.177 | 0.238 | 0.002 | 0.105 | 0.239 |
| Cd – Cr | 0.082 | 0.480 | 0.111 | 0.269 | 0.030 | 0.747 |
| Cd – Cu | -0.111 | 0.305 | -0.028 | 0.853 | 0.083 | 0.370 |
| Cd – Fe | -0.008 | 0.949 | -0.069 | 0.554 | 0.061 | 0.508 |
| Cd – Mg | -0.103 | 0.345 | -0.004 | 0.986 | 0.099 | 0.281 |
| Cd – Mn | 0.273 | <0.001 | 0.157 | 0.083 | 0.116 | 0.187 |
| Cd – Mo | 0.131 | 0.182 | 0.051 | 0.733 | 0.081 | 0.379 |
| Cd – Pb | 0.293 | <0.001 | 0.271 | <0.001 | 0.022 | 0.797 |
| Cd – Sb | -0.033 | 0.820 | -0.031 | 0.835 | 0.002 | 0.983 |
| Cd – Se | -0.113 | 0.295 | -0.111 | 0.269 | 0.003 | 0.975 |
| Cd – V | -0.180 | 0.034 | -0.035 | 0.821 | 0.145 | 0.114 |
| Cd – Zn | -0.073 | 0.549 | -0.035 | 0.830 | 0.038 | 0.684 |
| Co – As | -0.026 | 0.836 | 0.095 | 0.339 | 0.121 | 0.191 |
| Co – Ba | -0.028 | 0.841 | -0.224 | 0.004 | 0.196 | 0.031 |
| Co – Ca | 0.020 | 0.864 | 0.027 | 0.861 | 0.007 | 0.943 |
| Co – Cd | 0.133 | 0.177 | 0.238 | 0.002 | 0.105 | 0.239 |
| Co – Cr | 0.287 | <0.001 | 0.285 | <0.001 | 0.001 | 0.988 |
| Co – Cu | 0.083 | 0.474 | -0.035 | 0.814 | 0.118 | 0.202 |
| Co – Fe | -0.031 | 0.808 | -0.012 | 0.977 | 0.019 | 0.835 |
| Co – Mg | -0.070 | 0.555 | 0.088 | 0.396 | 0.158 | 0.087 |
| Co – Mn | -0.053 | 0.659 | -0.149 | 0.109 | 0.096 | 0.294 |
| Co – Mo | 0.067 | 0.584 | 0.104 | 0.294 | 0.037 | 0.684 |
| Co – Pb | 0.058 | 0.660 | 0.112 | 0.291 | 0.054 | 0.556 |
| Co – Sb | 0.351 | <0.001 | 0.311 | <0.001 | 0.040 | 0.629 |
| Co – Se | -0.026 | 0.834 | -0.035 | 0.817 | 0.008 | 0.929 |
| Co – V | 0.117 | 0.262 | 0.266 | <0.001 | 0.149 | 0.094 |
| Co – Zn | -0.066 | 0.588 | -0.032 | 0.834 | 0.034 | 0.714 |
| Cr – As | 0.014 | 0.918 | -0.053 | 0.706 | 0.067 | 0.468 |
| Cr – Ba | -0.083 | 0.483 | -0.037 | 0.834 | 0.046 | 0.618 |
| Cr – Ca | 0.057 | 0.661 | 0.112 | 0.284 | 0.055 | 0.548 |
| Cr – Cd | 0.082 | 0.480 | 0.111 | 0.269 | 0.030 | 0.747 |
| Cr – Co | 0.287 | <0.001 | 0.285 | <0.001 | 0.001 | 0.988 |
| Cr – Cu | -0.027 | 0.846 | 0.010 | 0.970 | 0.036 | 0.694 |
| Cr – Fe | -0.010 | 0.937 | 0.102 | 0.311 | 0.112 | 0.224 |
| Cr – Mg | -0.008 | 0.952 | -0.084 | 0.432 | 0.076 | 0.411 |
| Cr – Mn | 0.370 | <0.001 | 0.256 | 0.001 | 0.115 | 0.168 |
| Cr – Mo | 0.192 | 0.020 | 0.039 | 0.824 | 0.153 | 0.093 |
| Cr – Pb | 0.009 | 0.945 | 0.065 | 0.591 | 0.056 | 0.546 |
| Cr – Sb | 0.211 | 0.009 | 0.333 | <0.001 | 0.123 | 0.152 |
| Cr – Se | -0.005 | 0.961 | -0.019 | 0.933 | 0.015 | 0.873 |
| Cr – V | 0.148 | 0.110 | 0.076 | 0.489 | 0.072 | 0.429 |
| Cr – Zn | -0.150 | 0.105 | 0.006 | 0.991 | 0.156 | 0.089 |
| Cu – As | 0.094 | 0.410 | 0.010 | 0.992 | 0.084 | 0.362 |
| Cu – Ba | 0.020 | 0.858 | -0.016 | 0.937 | 0.035 | 0.704 |
| Cu – Ca | 0.372 | <0.001 | 0.339 | <0.001 | 0.033 | 0.685 |
| Cu – Cd | -0.111 | 0.305 | -0.028 | 0.853 | 0.083 | 0.370 |
| Cu – Co | 0.083 | 0.474 | -0.035 | 0.814 | 0.118 | 0.202 |
| Cu – Cr | -0.027 | 0.846 | 0.010 | 0.970 | 0.036 | 0.694 |
| Cu – Fe | -0.205 | 0.011 | -0.227 | 0.003 | 0.022 | 0.801 |
| Cu – Mg | 0.113 | 0.294 | 0.315 | <0.001 | 0.202 | 0.022 |
| Cu – Mn | -0.041 | 0.749 | 0.005 | 0.985 | 0.047 | 0.615 |
| Cu – Mo | 0.103 | 0.342 | 0.118 | 0.253 | 0.015 | 0.870 |
| Cu – Pb | -0.058 | 0.653 | -0.104 | 0.295 | 0.046 | 0.618 |
| Cu – Sb | -0.088 | 0.455 | -0.080 | 0.463 | 0.008 | 0.928 |
| Cu – Se | 0.185 | 0.026 | 0.010 | 0.983 | 0.175 | 0.056 |
| Cu – V | 0.089 | 0.464 | 0.050 | 0.735 | 0.039 | 0.672 |
| Cu – Zn | 0.100 | 0.354 | 0.125 | 0.205 | 0.025 | 0.786 |
| Fe – As | 0.007 | 0.940 | -0.043 | 0.784 | 0.050 | 0.587 |
| Fe – Ba | 0.156 | 0.089 | 0.112 | 0.277 | 0.044 | 0.631 |
| Fe – Ca | 0.075 | 0.521 | 0.112 | 0.271 | 0.037 | 0.691 |
| Fe – Cd | -0.008 | 0.949 | -0.069 | 0.554 | 0.061 | 0.508 |
| Fe – Co | -0.031 | 0.808 | -0.012 | 0.977 | 0.019 | 0.835 |
| Fe – Cr | -0.010 | 0.937 | 0.102 | 0.311 | 0.112 | 0.224 |
| Fe – Cu | -0.205 | 0.011 | -0.227 | 0.003 | 0.022 | 0.801 |
| Fe – Mg | -0.024 | 0.843 | -0.062 | 0.612 | 0.038 | 0.681 |
| Fe – Mn | 0.027 | 0.841 | 0.041 | 0.797 | 0.015 | 0.875 |
| Fe – Mo | -0.034 | 0.812 | -0.018 | 0.926 | 0.017 | 0.858 |
| Fe – Pb | 0.179 | 0.035 | 0.129 | 0.200 | 0.050 | 0.581 |
| Fe – Sb | 0.137 | 0.167 | 0.190 | 0.020 | 0.053 | 0.558 |
| Fe – Se | 0.054 | 0.645 | 0.134 | 0.180 | 0.079 | 0.387 |
| Fe – V | 0.020 | 0.860 | -0.044 | 0.789 | 0.064 | 0.491 |
| Fe – Zn | 0.247 | 0.001 | 0.137 | 0.161 | 0.110 | 0.218 |
| Mg – As | -0.012 | 0.923 | 0.044 | 0.787 | 0.056 | 0.542 |
| Mg – Ba | -0.052 | 0.654 | -0.100 | 0.306 | 0.048 | 0.605 |
| Mg – Ca | 0.323 | <0.001 | 0.308 | <0.001 | 0.015 | 0.859 |
| Mg – Cd | -0.103 | 0.345 | -0.004 | 0.986 | 0.099 | 0.281 |
| Mg – Co | -0.070 | 0.555 | 0.088 | 0.396 | 0.158 | 0.087 |
| Mg – Cr | -0.008 | 0.952 | -0.084 | 0.432 | 0.076 | 0.411 |
| Mg – Cu | 0.113 | 0.294 | 0.315 | <0.001 | 0.202 | 0.022 |
| Mg – Fe | -0.024 | 0.843 | -0.062 | 0.612 | 0.038 | 0.681 |
| Mg – Mn | 0.071 | 0.559 | 0.036 | 0.831 | 0.035 | 0.705 |
| Mg – Mo | 0.065 | 0.591 | 0.246 | 0.001 | 0.181 | 0.045 |
| Mg – Pb | -0.086 | 0.465 | 0.042 | 0.791 | 0.128 | 0.167 |
| Mg – Sb | -0.077 | 0.506 | -0.107 | 0.284 | 0.030 | 0.748 |
| Mg – Se | 0.206 | 0.010 | 0.004 | 0.979 | 0.202 | 0.027 |
| Mg – V | -0.136 | 0.164 | 0.114 | 0.282 | 0.250 | 0.007 |
| Mg – Zn | 0.084 | 0.477 | 0.139 | 0.151 | 0.055 | 0.547 |
| Mn – As | -0.078 | 0.503 | -0.018 | 0.925 | 0.059 | 0.521 |
| Mn – Ba | 0.040 | 0.752 | 0.358 | <0.001 | 0.318 | <0.001 |
| Mn – Ca | 0.089 | 0.463 | 0.067 | 0.565 | 0.022 | 0.815 |
| Mn – Cd | 0.273 | <0.001 | 0.157 | 0.083 | 0.116 | 0.187 |
| Mn – Co | -0.053 | 0.659 | -0.149 | 0.109 | 0.096 | 0.294 |
| Mn – Cr | 0.370 | <0.001 | 0.256 | 0.001 | 0.115 | 0.168 |
| Mn – Cu | -0.041 | 0.749 | 0.005 | 0.985 | 0.047 | 0.615 |
| Mn – Fe | 0.027 | 0.841 | 0.041 | 0.797 | 0.015 | 0.875 |
| Mn – Mg | 0.071 | 0.559 | 0.036 | 0.831 | 0.035 | 0.705 |
| Mn – Mo | 0.126 | 0.211 | -0.024 | 0.878 | 0.150 | 0.104 |
| Mn – Pb | 0.226 | 0.004 | 0.224 | 0.004 | 0.003 | 0.976 |
| Mn – Sb | -0.263 | <0.001 | -0.026 | 0.865 | 0.237 | 0.009 |
| Mn – Se | -0.032 | 0.817 | -0.019 | 0.932 | 0.013 | 0.885 |
| Mn – V | -0.079 | 0.499 | -0.252 | 0.001 | 0.172 | 0.055 |
| Mn – Zn | 0.051 | 0.670 | 0.045 | 0.786 | 0.006 | 0.952 |
| Mo – As | 0.022 | 0.859 | -0.010 | 0.977 | 0.032 | 0.729 |
| Mo – Ba | 0.013 | 0.920 | -0.101 | 0.306 | 0.114 | 0.217 |
| Mo – Ca | 0.003 | 0.964 | 0.076 | 0.485 | 0.073 | 0.427 |
| Mo – Cd | 0.131 | 0.182 | 0.051 | 0.733 | 0.081 | 0.379 |
| Mo – Co | 0.067 | 0.584 | 0.104 | 0.294 | 0.037 | 0.684 |
| Mo – Cr | 0.192 | 0.020 | 0.039 | 0.824 | 0.153 | 0.093 |
| Mo – Cu | 0.103 | 0.342 | 0.118 | 0.253 | 0.015 | 0.870 |
| Mo – Fe | -0.034 | 0.812 | -0.018 | 0.926 | 0.017 | 0.858 |
| Mo – Mg | 0.065 | 0.591 | 0.246 | 0.001 | 0.181 | 0.045 |
| Mo – Mn | 0.126 | 0.211 | -0.024 | 0.878 | 0.150 | 0.104 |
| Mo – Pb | 0.033 | 0.819 | -0.007 | 0.989 | 0.040 | 0.665 |
| Mo – Sb | 0.004 | 0.958 | -0.053 | 0.711 | 0.058 | 0.534 |
| Mo – Se | -0.020 | 0.872 | 0.095 | 0.344 | 0.116 | 0.211 |
| Mo – V | 0.031 | 0.816 | 0.105 | 0.296 | 0.073 | 0.426 |
| Mo – Zn | 0.108 | 0.304 | 0.109 | 0.272 | 0.001 | 0.992 |
| Pb – As | 0.132 | 0.182 | 0.072 | 0.527 | 0.060 | 0.511 |
| Pb – Ba | 0.345 | <0.001 | 0.224 | 0.004 | 0.120 | 0.157 |
| Pb – Ca | -0.087 | 0.453 | -0.126 | 0.212 | 0.038 | 0.675 |
| Pb – Cd | 0.293 | <0.001 | 0.271 | <0.001 | 0.022 | 0.797 |
| Pb – Co | 0.058 | 0.660 | 0.112 | 0.291 | 0.054 | 0.556 |
| Pb – Cr | 0.009 | 0.945 | 0.065 | 0.591 | 0.056 | 0.546 |
| Pb – Cu | -0.058 | 0.653 | -0.104 | 0.295 | 0.046 | 0.618 |
| Pb – Fe | 0.179 | 0.035 | 0.129 | 0.200 | 0.050 | 0.581 |
| Pb – Mg | -0.086 | 0.465 | 0.042 | 0.791 | 0.128 | 0.167 |
| Pb – Mn | 0.226 | 0.004 | 0.224 | 0.004 | 0.003 | 0.976 |
| Pb – Mo | 0.033 | 0.819 | -0.007 | 0.989 | 0.040 | 0.665 |
| Pb – Sb | -0.055 | 0.661 | 0.003 | 0.983 | 0.058 | 0.530 |
| Pb – Se | 0.007 | 0.947 | -0.017 | 0.934 | 0.024 | 0.799 |
| Pb – V | -0.055 | 0.650 | 0.054 | 0.712 | 0.109 | 0.238 |
| Pb – Zn | 0.054 | 0.650 | 0.081 | 0.463 | 0.026 | 0.776 |
| Sb – As | -0.062 | 0.629 | 0.005 | 0.981 | 0.067 | 0.470 |
| Sb – Ba | 0.019 | 0.863 | -0.162 | 0.069 | 0.180 | 0.050 |
| Sb – Ca | 0.099 | 0.358 | 0.129 | 0.197 | 0.030 | 0.742 |
| Sb – Cd | -0.033 | 0.820 | -0.031 | 0.835 | 0.002 | 0.983 |
| Sb – Co | 0.351 | <0.001 | 0.311 | <0.001 | 0.040 | 0.629 |
| Sb – Cr | 0.211 | 0.009 | 0.333 | <0.001 | 0.123 | 0.152 |
| Sb – Cu | -0.088 | 0.455 | -0.080 | 0.463 | 0.008 | 0.928 |
| Sb – Fe | 0.137 | 0.167 | 0.190 | 0.020 | 0.053 | 0.558 |
| Sb – Mg | -0.077 | 0.506 | -0.107 | 0.284 | 0.030 | 0.748 |
| Sb – Mn | -0.263 | <0.001 | -0.026 | 0.865 | 0.237 | 0.009 |
| Sb – Mo | 0.004 | 0.958 | -0.053 | 0.711 | 0.058 | 0.534 |
| Sb – Pb | -0.055 | 0.661 | 0.003 | 0.983 | 0.058 | 0.530 |
| Sb – Se | 0.046 | 0.721 | 0.100 | 0.310 | 0.055 | 0.552 |
| Sb – V | -0.056 | 0.661 | -0.020 | 0.931 | 0.036 | 0.697 |
| Sb – Zn | 0.028 | 0.846 | 0.038 | 0.826 | 0.010 | 0.915 |
| Se – As | 0.295 | <0.001 | 0.115 | 0.275 | 0.180 | 0.042 |
| Se – Ba | 0.045 | 0.709 | -0.002 | 0.984 | 0.047 | 0.611 |
| Se – Ca | 0.349 | <0.001 | 0.191 | 0.020 | 0.158 | 0.065 |
| Se – Cd | -0.113 | 0.295 | -0.111 | 0.269 | 0.003 | 0.975 |
| Se – Co | -0.026 | 0.834 | -0.035 | 0.817 | 0.008 | 0.929 |
| Se – Cr | -0.005 | 0.961 | -0.019 | 0.933 | 0.015 | 0.873 |
| Se – Cu | 0.185 | 0.026 | 0.010 | 0.983 | 0.175 | 0.056 |
| Se – Fe | 0.054 | 0.645 | 0.134 | 0.180 | 0.079 | 0.387 |
| Se – Mg | 0.206 | 0.010 | 0.004 | 0.979 | 0.202 | 0.027 |
| Se – Mn | -0.032 | 0.817 | -0.019 | 0.932 | 0.013 | 0.885 |
| Se – Mo | -0.020 | 0.872 | 0.095 | 0.344 | 0.116 | 0.211 |
| Se – Pb | 0.007 | 0.947 | -0.017 | 0.934 | 0.024 | 0.799 |
| Se – Sb | 0.046 | 0.721 | 0.100 | 0.310 | 0.055 | 0.552 |
| Se – V | -0.069 | 0.566 | -0.005 | 0.993 | 0.063 | 0.493 |
| Se – Zn | 0.206 | 0.011 | 0.171 | 0.048 | 0.036 | 0.690 |
| V – As | 0.046 | 0.715 | 0.094 | 0.343 | 0.048 | 0.600 |
| V – Ba | 0.111 | 0.301 | 0.010 | 0.962 | 0.101 | 0.275 |
| V – Ca | -0.035 | 0.801 | 0.087 | 0.403 | 0.122 | 0.187 |
| V – Cd | -0.180 | 0.034 | -0.035 | 0.821 | 0.145 | 0.114 |
| V – Co | 0.117 | 0.262 | 0.266 | <0.001 | 0.149 | 0.094 |
| V – Cr | 0.148 | 0.110 | 0.076 | 0.489 | 0.072 | 0.429 |
| V – Cu | 0.089 | 0.464 | 0.050 | 0.735 | 0.039 | 0.672 |
| V – Fe | 0.020 | 0.860 | -0.044 | 0.789 | 0.064 | 0.491 |
| V – Mg | -0.136 | 0.164 | 0.114 | 0.282 | 0.250 | 0.007 |
| V – Mn | -0.079 | 0.499 | -0.252 | 0.001 | 0.172 | 0.055 |
| V – Mo | 0.031 | 0.816 | 0.105 | 0.296 | 0.073 | 0.426 |
| V – Pb | -0.055 | 0.650 | 0.054 | 0.712 | 0.109 | 0.238 |
| V – Sb | -0.056 | 0.661 | -0.020 | 0.931 | 0.036 | 0.697 |
| V – Se | -0.069 | 0.566 | -0.005 | 0.993 | 0.063 | 0.493 |
| V – Zn | -0.036 | 0.802 | 0.059 | 0.652 | 0.095 | 0.306 |
| Zn – As | -0.023 | 0.852 | -0.032 | 0.829 | 0.009 | 0.927 |
| Zn – Ba | 0.043 | 0.728 | 0.033 | 0.833 | 0.010 | 0.910 |
| Zn – Ca | 0.366 | <0.001 | 0.366 | <0.001 | 0.001 | 0.998 |
| Zn – Cd | -0.073 | 0.549 | -0.035 | 0.830 | 0.038 | 0.684 |
| Zn – Co | -0.066 | 0.588 | -0.032 | 0.834 | 0.034 | 0.714 |
| Zn – Cr | -0.150 | 0.105 | 0.006 | 0.991 | 0.156 | 0.089 |
| Zn – Cu | 0.100 | 0.354 | 0.125 | 0.205 | 0.025 | 0.786 |
| Zn – Fe | 0.247 | 0.001 | 0.137 | 0.161 | 0.110 | 0.218 |
| Zn – Mg | 0.084 | 0.477 | 0.139 | 0.151 | 0.055 | 0.547 |
| Zn – Mn | 0.051 | 0.670 | 0.045 | 0.786 | 0.006 | 0.952 |
| Zn – Mo | 0.108 | 0.304 | 0.109 | 0.272 | 0.001 | 0.992 |
| Zn – Pb | 0.054 | 0.650 | 0.081 | 0.463 | 0.026 | 0.776 |
| Zn – Sb | 0.028 | 0.846 | 0.038 | 0.826 | 0.010 | 0.915 |
| Zn – Se | 0.206 | 0.011 | 0.171 | 0.048 | 0.036 | 0.690 |
| Zn – V | -0.036 | 0.802 | 0.059 | 0.652 | 0.095 | 0.306 |

Analysis is based on a spearman’s rho rank correlation between the different trace elements and adjusted for multiple testing using FDR correction. P values that were statistical significant after FDR correction (P_FDR_-value <0.05) are highlighted in grey boxes. Correlation coefficients of nAMD patients and controls were compared using the “r. test” function of the “psych” package. Abbreviations: As, arsenic; Ba, barium; Ca, calcium; Cd, cadmium; Co, cobalt; Cr, chromium; Cu, copper; Fe, iron; LOQ: limit of quantification; Mg, magnesium; Mn, manganese; Mo, molybdenum; nAMD, neovascular age-related macular degeneration; Pb, lead; Sb, antimony; Se, selenium; V, vanadium; Zn, zinc.

**Supplementary Table 3.** Association between plasma trace element levels in non-AMD controls and lens status

|  |  | **As**  **[µg/L]** | **Ba [µg/L]** | **Ca**  **[µg/L]** | **Cd [µg/L]** | **Co [µg/L]** | **Cr [µg/L]** | **Cu**  **[µg/L]** | **Fe**  **[µg/L]** | **Mg [µg/L]** | **Mn [µg/L]** | **Mo [µg/L]** | **Pb [µg/L]** | **Sb [µg/L]** | **Se [µg/L]** | **V [µg/L]** | **Zn [µg/L]** |
| --- | --- | --- | --- | --- | --- | --- | --- | --- | --- | --- | --- | --- | --- | --- | --- | --- | --- |
| Phakic, no cataract [n=97(56%] |  | 0.424 | 1.185 | 88538.4 | 0.008 | 0.170 | 0.428 | 922.3 | 1179.5 | 18252.6 | 0.909 | 0.892 | 0.061 | 2.580 | 91.78 | 0.093 | 752.2 |
| Phakic, cataract [n=45 (26%)] |  | 0.440 | 1.269 | 88317.1 | 0.014 | 0.157 | 0.427 | 914.1 | 1146.4 | 18969.5 | 0.959 | 0.938 | 0.050 | 2.598 | 93.85 | 0.090 | 753.4 |
| Pseudophakic [n=32 (18%)] |  | 0.408 | 1.106 | 87758.4 | 0.012 | 0.131 | 0.465 | 906.7 | 1128.2 | 17940.9 | 0.900 | 1.000 | 0.083 | 2.446 | 91.41 | 0.092 | 803.9 |
|  | **P value** | **0.399** | **0.561** | **0.766** | **0.573** | **0.341** | **0.880** | **0.889** | **0.682** | **0.034** | **0.897** | **0.659** | **0.717** | **0.689** | **0.723** | **0.968** | **0.045** |

Analyses are based on ANOVA t-tests for normally distributed trace elements, and Kruskal-Wallis H tests for non-normally distributed trace elements. There were no significant differences in plasma trace element levels between lens status categories after applying Bonferroni correction for 16 covariates (the Bonferroni adjusted threshold for statistical significance was defined as P<0.003). Abbreviations: ANOVA, Analysis of variance; As, arsenic; Ba, barium; Ca, calcium; Cd, cadmium; Co, cobalt; Cr, chromium; Cu, copper; Fe, iron; Mg, magnesium; Mn, manganese; Mo, molybdenum; Pb, lead; Sb, antimony; Se, selenium; V, vanadium; Zn, zinc.

**Supplementary Table 4**. Association between plasma trace element levels and genetic loci associated with AMD

|  |  | **As**  **[µg/L]** | **Ba [µg/L]** | **Ca**  **[µg/L]** | **Cd [µg/L]** | **Co [µg/L]** | **Cr [µg/L]** | **Cu**  **[µg/L]** | **Fe**  **[µg/L]** | **Mg [µg/L]** | **Mn [µg/L]** | **Mo [µg/L]** | **Pb [µg/L]** | **Sb [µg/L]** | **Se [µg/L]** | **V [µg/L]** | **Zn [µg/L]** |
| --- | --- | --- | --- | --- | --- | --- | --- | --- | --- | --- | --- | --- | --- | --- | --- | --- | --- |
| *CFH* rs10922109 |  |  |  |  |  |  |  |  |  |  |  |  |  |  |  |  |  |
| C/C [n=210 (50%)] |  | 0.435 | 1.281 | 89503.2 | 0.013 | 0.137 | 0.402 | 967.3 | 1128.6 | 18582.3 | 0.949 | 0.854 | 0.053 | 2.480 | 92.48 | 0.088 | 774.9 |
| C/A [n=170 (40%)] |  | 0.431 | 1.163 | 87999.2 | 0.014 | 0.151 | 0.420 | 922.5 | 1160.7 | 18676.3 | 0.952 | 0.977 | 0.061 | 2.489 | 92.78 | 0.086 | 755.4 |
| A/A [n=41 (10%)] |  | 0.432 | 1.264 | 88825.3 | 0.015 | 0.173 | 0.506 | 910.4 | 1330.5 | 18305.0 | 0.915 | 0.843 | 0.100 | 2.631 | 92.89 | 0.104 | 774.7 |
|  | **P value** | **0.773** | **0.202** | **0.157** | **0.443** | **0.165** | **0.126** | **0.025** | **0.012** | **0.541** | **0.945** | **0.034** | **0.572** | **0.506** | **0.980** | **0.099** | **0.248** |
| *CFH* rs570618 |  |  |  |  |  |  |  |  |  |  |  |  |  |  |  |  |  |
| T/T [n=124 (30%)] |  | 0.448 | 1.232 | 88630.5 | 0.012 | 0.137 | 0.389 | 945.9 | 1117.5 | 18659.8 | 0.900 | 0.859 | 0.050 | 2.494 | 92.47 | 0.087 | 772.3 |
| T/G [n=191 (45%)] |  | 0.437 | 1.198 | 88955.4 | 0.014 | 0.149 | 0.427 | 947.5 | 1147.2 | 18530.4 | 0.952 | 0.915 | 0.102 | 2.499 | 91.98 | 0.090 | 760.0 |
| G/G [n=107 (25%)] |  | 0.411 | 1.296 | 88806.5 | 0.016 | 0.155 | 0.440 | 933.5 | 1232.0 | 18635.0 | 0.990 | 0.938 | 0.082 | 2.494 | 93.96 | 0.089 | 772.2 |
|  | **P value** | **0.817** | **0.460** | **0.933** | **0.333** | **0.506** | **0.386** | **0.800** | **0.078** | **0.820** | **0.565** | **0.434** | **0.452** | **0.997** | **0.625** | **0.820** | **0.565** |
| *COL4A3* rs11884770 |  |  |  |  |  |  |  |  |  |  |  |  |  |  |  |  |  |
| C/C [n=224 (53%)] |  | 0.839 | 1.239 | 88791.8 | 0.014 | 0.141 | 0.411 | 941.9 | 1150.8 | 18552.8 | 0.905 | 0.904 | 0.055 | 2.492 | 92.36 | 0.092 | 763.4 |
| T/C [n=159 (38%)] |  | 0.704 | 1.215 | 88823.4 | 0.015 | 0.153 | 0.444 | 942.7 | 1196.1 | 18678.4 | 1.004 | 0.903 | 0.056 | 2.540 | 92.53 | 0.087 | 770.1 |
| T/T [n=38 (9%)] |  | 1.042 | 1.253 | 89081.2 | 0.013 | 0.147 | 0.365 | 958.4 | 1081.1 | 18475.4 | 0.954 | 0.897 | 0.136 | 2.366 | 94.79 | 0.081 | 775.1 |
|  | **P value** | **0.222** | **0.917** | **0.977** | **0.573** | **0.618** | **0.292** | **0.869** | **0.234** | **0.761** | **0.343** | **0.996** | **0.484** | **0.447** | **0.721** | **0.323** | **0.780** |
| *ADAMTS9-AS2* rs62247658 |  |  |  |  |  |  |  |  |  |  |  |  |  |  |  |  |  |
| T/T [n=150 (36%)] |  | 0.846 | 1.261 | 88761.1 | 0.015 | 0.144 | 0.437 | 952.9 | 1177.1 | 18621.3 | 0.930 | 0.912 | 0.102 | 2.498 | 94.59 | 0.092 | 774.3 |
| C/T [n=177 (42%)] |  | 0.708 | 1.213 | 88457.5 | 0.013 | 0.146 | 0.410 | 943.7 | 1128.3 | 18517.0 | 0.924 | 0.859 | 0.050 | 2.527 | 91.71 | 0.086 | 762.4 |
| C/C [n=92 (22%)] |  | 0.947 | 1.237 | 89623.6 | 0.014 | 0.153 | 0.406 | 929.8 | 1194.9 | 18630.2 | 1.013 | 0.982 | 0.087 | 2.434 | 91.69 | 0.090 | 762.7 |
|  | **P value** | **0.127** | **0.806** | **0.487** | **0.597** | **0.844** | **0.640** | **0.627** | **0.353** | **0.853** | **0.533** | **0.141** | **0.236** | **0.642** | **0.253** | **0.584** | **0.617** |
| *COL8A1* *rs140647181* |  |  |  |  |  |  |  |  |  |  |  |  |  |  |  |  |  |
| T/T [n=402 (96%)] |  | 0.811 | 1.237 | 88964.3 | 0.014 | 0.148 | 0.423 | 945.3 | 1159.5 | 18606.5 | 0.945 | 0.905 | 0.055 | 2.502 | 92.70 | 0.090 | 766.6 |
| T/C or C/C [n=18 (4%)] |  | 0.734 | 1.162 | 85968.9 | 0.022 | 0.123 | 0.351 | 890.0 | 1190.1 | 18418.6 | 1.043 | 0.862 | 0.138 | 2.274 | 93.86 | 0.062 | 769.1 |
|  | **P value** | **0.426** | **0.635** | **0.101** | **0.129** | **0.384** | **0.317** | **0.202** | **0.750** | **0.687** | **0.528** | **0.713** | **0.245** | **0.219** | **0.777** | **0.019** | **0.930** |
| *CFI* rs10033900 |  |  |  |  |  |  |  |  |  |  |  |  |  |  |  |  |  |
| T/T [n=108 (26%)] |  | 0.408 | 1.235 | 89052.1 | 0.014 | 0.142 | 0.426 | 970.2 | 1142.4 | 18888.6 | 0.917 | 0.876 | 0.050 | 2.538 | 94.35 | 0.089 | 760.3 |
| T/C [n=205 (48%)] |  | 0.456 | 1.188 | 88847.1 | 0.015 | 0.148 | 0.404 | 934.0 | 1183.6 | 18545.6 | 0.956 | 0.917 | 0.068 | 2.442 | 91.44 | 0.089 | 759.8 |
| C/C [n=110 (26%)] |  | 0.408 | 1.312 | 88452.7 | 0.013 | 0.151 | 0.443 | 933.3 | 1134.6 | 18400.8 | 0.958 | 0.908 | 0.075 | 2.552 | 93.03 | 0.089 | 785.8 |
|  | **P value** | **0.701** | **0.277** | **0.837** | **0.756** | **0.859** | **0.533** | **0.193** | **0.506** | **0.153** | **0.862** | **0.771** | **0.188** | **0.389** | **0.342** | **0.998** | **0.139** |
| *C9* *rs62358361* |  |  |  |  |  |  |  |  |  |  |  |  |  |  |  |  |  |
| G/G [n=414 (98%)] |  | 0.806 | 1.236 | 88782.2 | 0.014 | 0.147 | 0.415 | 939.9 | 1162.9 | 18608.5 | 0.939 | 0.902 | 0.063 | 2.494 | 92.59 | 0.089 | 768.0 |
| G/T or T/T [n=9 (2%)] |  | 0.702 | 1.063 | 89472.3 | 0.019 | 0.150 | 0.609 | 1090.1 | 1037.7 | 18000.5 | 1.300 | 1.014 | 0.025 | 2.523 | 93.04 | 0.070 | 707.5 |
|  | **P value** | **0.975** | **0.430** | **0.787** | **0.297** | **0.948** | **0.054** | **0.013** | **0.351** | **0.350** | **0.098** | **0.491** | **0.151** | **0.912** | **0.937** | **0.242** | **0.126** |
| *PRLR/SPEF2* rs74767144* |  |  |  |  |  |  |  |  |  |  |  |  |  |  |  |  |  |
| C/C [n=399 (95%)] |  | 0.803 | 1.219 | 88834.5 | 0.014 | 0.149 | 0.422 | 941.5 | 1162.3 | 18597.1 | 0.955 | 0.899 | 0.062 | 2.487 | 92.51 | 0.088 | 766.1 |
| C/G or G/G [n=23 (5%)] |  | 0.823 | 1.368 | 88334.7 | 0.013 | 0.127 | 0.383 | 984.5 | 1132.1 | 18529.8 | 0.802 | 1.002 | 0.050 | 2.644 | 94.29 | 0.097 | 778.0 |
|  | **P value** | **0.222** | **0.284** | **0.759** | **0.556** | **0.402** | **0.549** | **0.264** | **0.724** | **0.871** | **0.283** | **0.326** | **0.694** | **0.343** | **0.635** | **0.433** | **0.637** |
| *C2/CFB/SKIV2L* rs116503776 |  |  |  |  |  |  |  |  |  |  |  |  |  |  |  |  |  |
| G/G [n=380 (91%)] |  | 0.431 | 1.236 | 88887.3 | 0.014 | 0.150 | 0.419 | 946.2 | 1146.9 | 18584.3 | 0.931 | 0.906 | 0.074 | 2.519 | 92.77 | 0.089 | 770.0 |
| G/A or A/A [n=40 (9%)] |  | 0.396 | 1.215 | 88157.1 | 0.015 | 0.113 | 0.427 | 909.2 | 1288.2 | 18615.2 | 1.112 | 0.895 | 0.050 | 2.311 | 90.71 | 0.090 | 730.7 |
|  | **P value** | **0.529** | **0.851** | **0.562** | **0.993** | **0.058** | **0.871** | **0.216** | **0.033** | **0.923** | **0.095** | **0.889** | **0.295** | **0.104** | **0.468** | **0.872** | **0.042** |
| *VEGFA* rs943080 |  |  |  |  |  |  |  |  |  |  |  |  |  |  |  |  |  |
| T/T [n=128 (30%)] |  | 0.384 | 1.158 | 89468.6 | 0.012 | 0.153 | 0.393 | 931.8 | 1150.7 | 18674.2 | 0.825 | 0.914 | 0.050 | 2.516 | 92.69 | 0.092 | 758.7 |
| C/T [n=192 (46%)] |  | 0.440 | 1.299 | 88077.8 | 0.015 | 0.148 | 0.430 | 948.6 | 1158.8 | 18567.9 | 0.957 | 0.897 | 0.075 | 2.483 | 92.63 | 0.089 | 761.7 |
| C/C [n=100 (24%)] |  | 0.444 | 1.166 | 89536.6 | 0.015 | 0.141 | 0.429 | 953.3 | 1164.4 | 18561.9 | 1.070 | 0.911 | 0.104 | 2.476 | 92.76 | 0.085 | 787.9 |
|  | **P value** | **0.956** | **0.091** | **0.160** | **0.245** | **0.758** | **0.506** | **0.611** | **0.966** | **0.869** | **0.017** | **0.945** | **0.044** | **0.911** | **0.998** | **0.611** | **0.123** |
| *KMT2E/SRPK2* rs1142 |  |  |  |  |  |  |  |  |  |  |  |  |  |  |  |  |  |
| C/C [n=143 (34%)] |  | 0.734 | 1.233 | 89481.3 | 0.014 | 0.145 | 0.480 | 954.9 | 1145.2 | 18660.9 | 1.008 | 0.900 | 0.050 | 2.465 | 89.99 | 0.083 | 765.3 |
| C/T [n=210 (50%)] |  | 0.848 | 1.206 | 88318.9 | 0.015 | 0.154 | 0.388 | 938.8 | 1168.2 | 18467.0 | 0.869 | 0.908 | 0.061 | 2.532 | 93.99 | 0.093 | 761.7 |
| T/T [n=65 (16%)] |  | 0.839 | 1.309 | 88710.0 | 0.013 | 0.135 | 0.397 | 938.1 | 1149.6 | 18803.9 | 1.071 | 0.904 | 0.133 | 2.478 | 93.13 | 0.086 | 787.8 |
|  | **P value** | **0.048** | **0.537** | **0.371** | **0.193** | **0.530** | **0.015** | **0.680** | **0.858** | **0.402** | **0.037** | **0.989** | **0.074** | **0.704** | **0.089** | **0.189** | **0.291** |
| *PILRB/PILRA* rs7803454 |  |  |  |  |  |  |  |  |  |  |  |  |  |  |  |  |  |
| C/C [n=268 (64%)] |  | 0.874 | 1.197 | 89170.2 | 0.015 | 0.148 | 0.422 | 945.5 | 1133.3 | 18691.3 | 0.933 | 0.866 | 0.061 | 2.445 | 93.62 | 0.092 | 764.4 |
| C/T [n=136 (33%)] |  | 0.719 | 1.253 | 88392.1 | 0.013 | 0.143 | 0.404 | 948.3 | 1198.5 | 18442.5 | 0.960 | 0.993 | 0.052 | 2.606 | 90.87 | 0.082 | 768.1 |
| T/T [n=14 (3%)] |  | 0.445 | 1.582 | 87678.8 | 0.018 | 0.142 | 0.534 | 915.8 | 1385.2 | 18367.5 | 1.149 | 0.779 | 0.157 | 2.536 | 89.60 | 0.086 | 806.2 |
|  | **P value** | **0.141** | **0.182** | **0.081** | **0.331** | **0.522** | **0.895** | **0.295** | **0.813** | **0.043** | **0.429** | **0.468** | **0.165** | **0.028** | **0.141** | **0.137** | **0.428** |
| *TNFRSF10A* rs79037040 |  |  |  |  |  |  |  |  |  |  |  |  |  |  |  |  |  |
| T/T [n=123 (30%)] |  | 0.791 | 1.242 | 88701.3 | 0.014 | 0.148 | 0.420 | 956.1 | 1232.4 | 18808.9 | 0.996 | 0.874 | 0.109 | 2.588 | 93.43 | 0.101 | 765.4 |
| T/G [n=203 (48%)] |  | 0.837 | 1.256 | 89340.7 | 0.014 | 0.136 | 0.403 | 943.7 | 1128.4 | 18495.4 | 0.956 | 0.924 | 0.061 | 2.426 | 92.76 | 0.083 | 767.1 |
| G/G [n=94 (22%)] |  | 0.758 | 1.157 | 87741.0 | 0.014 | 0.164 | 0.459 | 927.3 | 1128.1 | 18528.0 | 0.868 | 0.901 | 0.050 | 2.539 | 91.02 | 0.082 | 768.4 |
|  | **P value** | **0.587** | **0.829** | **0.462** | **0.765** | **0.238** | **0.147** | **0.325** | **0.508** | **0.050** | **0.342** | **0.351** | **0.104** | **0.662** | **0.157** | **0.006** | **0.983** |
| *MIR6130/RORB* rs10781182 |  |  |  |  |  |  |  |  |  |  |  |  |  |  |  |  |  |
| G/G [n=195 (46%)] |  | 0.706 | 1.213 | 88935.5 | 0.014 | 0.141 | 0.407 | 939.3 | 1193.7 | 18598.0 | 0.936 | 0.947 | 0.061 | 2.480 | 91.70 | 0.085 | 763.8 |
| T/G [n=189 (45%)] |  | 0.928 | 1.236 | 88903.6 | 0.014 | 0.150 | 0.441 | 957.5 | 1117.6 | 18620.5 | 0.995 | 0.863 | 0.054 | 2.512 | 93.43 | 0.092 | 770.7 |
| T/T [n=38 (9%)] |  | 0.707 | 1.297 | 87639.2 | 0.015 | 0.153 | 0.376 | 893.1 | 1213.8 | 18439.5 | 0.766 | 0.872 | 0.136 | 2.514 | 93.17 | 0.093 | 764.4 |
|  | **P value** | **0.009** | **0.758** | **0.612** | **0.555** | **0.674** | **0.347** | **0.121** | **0.122** | **0.870** | **0.139** | **0.219** | **0.551** | **0.910** | **0.599** | **0.493** | **0.839** |
| *TRPM3* rs71507014 |  |  |  |  |  |  |  |  |  |  |  |  |  |  |  |  |  |
| GC/GC [n=115 (27%)] |  | 0.812 | 1.080 | 90246.8 | 0.014 | 0.131 | 0.385 | 953.6 | 1155.1 | 18778.4 | 0.904 | 0.887 | 0.050 | 2.471 | 92.64 | 0.084 | 776.9 |
| GC/G [n=211 (50%)] |  | 0.803 | 1.286 | 87951.1 | 0.014 | 0.155 | 0.432 | 934.4 | 1182.2 | 18463.1 | 0.916 | 0.915 | 0.090 | 2.540 | 92.26 | 0.090 | 754.3 |
| G/G [n=95 (23%)] |  | 0.806 | 1.299 | 88954.3 | 0.014 | 0.151 | 0.435 | 948.3 | 1111.8 | 18675.3 | 1.064 | 0.902 | 0.061 | 2.442 | 93.22 | 0.091 | 781.7 |
|  | **P value** | **0.706** | **0.013** | **0.032** | **0.865** | **0.214** | **0.340** | **0.619** | **0.356** | **0.337** | **0.133** | **0.877** | **0.757** | **0.530** | **0.901** | **0.135** | **0.091** |
| *TGFBR1* rs1626340 |  |  |  |  |  |  |  |  |  |  |  |  |  |  |  |  |  |
| G/G [n=275 (65%)] |  | 0.768 | 1.197 | 88766.7 | 0.014 | 0.152 | 0.411 | 941.2 | 1173.9 | 18672.5 | 0.918 | 0.906 | 0.072 | 2.530 | 92.27 | 0.089 | 770.0 |
| G/A [n=134 (32%)] |  | 0.903 | 1.290 | 88748.4 | 0.015 | 0.143 | 0.443 | 948.8 | 1130.4 | 18450.1 | 0.995 | 0.888 | 0.050 | 2.459 | 93.55 | 0.088 | 759.4 |
| A/A [n=14 (3%)] |  | 0.556 | 1.375 | 89853.3 | 0.013 | 0.090 | 0.362 | 924.9 | 1181.1 | 18475.7 | 1.043 | 1.016 | 0.061 | 2.145 | 89.89 | 0.094 | 772.1 |
|  | **P value** | **0.196** | **0.288** | **0.869** | **0.299** | **0.147** | **0.456** | **0.859** | **0.575** | **0.535** | **0.458** | **0.640** | **0.527** | **0.152** | **0.647** | **0.777** | **0.683** |
| *ABCA1* rs2740488 |  |  |  |  |  |  |  |  |  |  |  |  |  |  |  |  |  |
| A/A [n=215 (51%)] |  | 0.798 | 1.233 | 89523.4 | 0.014 | 0.150 | 0.426 | 958.6 | 1162.9 | 18623.4 | 0.999 | 0.890 | 0.050 | 2.518 | 92.12 | 0.087 | 763.8 |
| A/C [n=179 (42%)] |  | 0.833 | 1.197 | 88219.3 | 0.014 | 0.149 | 0.416 | 928.9 | 1143.3 | 18574.3 | 0.896 | 0.923 | 0.081 | 2.458 | 93.21 | 0.091 | 769.6 |
| C/C [n=28 (7%)] |  | 0.675 | 1.464 | 87291.5 | 0.015 | 0.114 | 0.385 | 921.0 | 1247.2 | 18508.5 | 0.875 | 0.897 | 0.147 | 2.568 | 92.74 | 0.089 | 770.1 |
|  | **P value** | **0.636** | **0.131** | **0.127** | **0.995** | **0.303** | **0.777** | **0.210** | **0.435** | **0.941** | **0.250** | **0.794** | **0.595** | **0.654** | **0.818** | **0.878** | **0.876** |
| *ARHGAP21* rs12357257 |  |  |  |  |  |  |  |  |  |  |  |  |  |  |  |  |  |
| G/G [n=267 (63%)] |  | 0.804 | 1.235 | 88998.6 | 0.014 | 0.145 | 0.425 | 935.3 | 1161.4 | 18656.3 | 0.970 | 0.910 | 0.072 | 2.486 | 93.26 | 0.091 | 766.5 |
| G/A [n=131 (31%)] |  | 0.841 | 1.206 | 88513.3 | 0.014 | 0.148 | 0.418 | 953.6 | 1162.3 | 18527.8 | 0.920 | 0.886 | 0.050 | 2.519 | 91.49 | 0.090 | 768.4 |
| A/A [n=24 (6%)] |  | 0.633 | 1.314 | 88234.0 | 0.016 | 0.143 | 0.370 | 975.5 | 1157.9 | 18259.2 | 0.839 | 0.918 | 0.050 | 2.505 | 91.47 | 0.060 | 763.6 |
|  | **P value** | **0.777** | **0.744** | **0.778** | **0.630** | **0.968** | **0.690** | **0.422** | **0.999** | **0.563** | **0.541** | **0.888** | **0.425** | **0.921** | **0.593** | **0.017** | **0.978** |
| *ARMS2/HTRA1* rs3750846 |  |  |  |  |  |  |  |  |  |  |  |  |  |  |  |  |  |
| T/T [n=181 (43%)] |  | 0.407 | 1.161 | 88691.8 | 0.014 | 0.153 | 0.469 | 928.9 | 1169.5 | 18284.1 | 0.954 | 0.930 | 0.061 | 2.522 | 92.01 | 0.090 | 754.6 |
| T/C [n=173 (41%)] |  | 0.441 | 1.292 | 88965.4 | 0.014 | 0.145 | 0.380 | 958.9 | 1164.1 | 18889.0 | 0.924 | 0.862 | 0.050 | 2.416 | 92.16 | 0.089 | 777.8 |
| C/C [n=67 (16%)] |  | 0.415 | 1.245 | 88870.3 | 0.013 | 0.138 | 0.385 | 947.9 | 1128.2 | 18662.2 | 0.987 | 0.948 | 0.142 | 2.634 | 95.61 | 0.086 | 771.3 |
|  | **P value** | **0.913** | **0.157** | **0.943** | **0.756** | **0.626** | **0.012** | **0.285** | **0.762** | **0.012** | **0.791** | **0.306** | **0.188** | **0.123** | **0.304** | **0.852** | **0.168** |
| *RDH5/CD63* rs3138141 |  |  |  |  |  |  |  |  |  |  |  |  |  |  |  |  |  |
| C/C [n=251 (60%)] |  | 0.896 | 1.233 | 88682.5 | 0.013 | 0.153 | 0.447 | 939.9 | 1131.1 | 18670.7 | 0.967 | 0.921 | 0.050 | 2.488 | 92.85 | 0.087 | 763.9 |
| C/A [n=153 (36%)] |  | 0.658 | 1.218 | 89104.9 | 0.015 | 0.134 | 0.385 | 954.5 | 1192.7 | 18503.7 | 0.920 | 0.889 | 0.101 | 2.511 | 91.60 | 0.093 | 769.6 |
| A/A [n=15 (4%)] |  | 0.795 | 1.385 | 87817.4 | 0.005 | 0.201 | 0.347 | 882.7 | 1286.4 | 18207.1 | 0.952 | 0.818 | 0.050 | 2.531 | 97.75 | 0.070 | 793.1 |
|  | **P value** | **0.974** | **0.640** | **0.758** | **0.558** | **0.066** | **0.080** | **0.302** | **0.145** | **0.517** | **0.786** | **0.632** | **0.484** | **0.944** | **0.369** | **0.384** | **0.612** |
| *ACAD10* rs61941272* |  |  |  |  |  |  |  |  |  |  |  |  |  |  |  |  |  |
| C/C [n=405 (96%)] |  | 0.812 | 1.225 | 88776.8 | 0.014 | 0.148 | 0.419 | 942.0 | 1164.4 | 18609.3 | 0.942 | 0.905 | 0.062 | 2.474 | 92.74 | 0.089 | 765.7 |
| C/A or A/A [n=18 (4%)] |  | 0.603 | 1.386 | 89249.2 | 0.014 | 0.136 | 0.426 | 967.0 | 1067.9 | 18284.6 | 1.057 | 0.897 | 0.050 | 2.976 | 89.35 | 0.085 | 788.2 |
|  | **P value** | **0.807** | **0.309** | **0.796** | **0.576** | **0.673** | **0.923** | **0.564** | **0.315** | **0.485** | **0.459** | **0.949** | **0.879** | **0.007** | **0.409** | **0.909** | **0.428** |
| *B3GALTL* rs9564692 |  |  |  |  |  |  |  |  |  |  |  |  |  |  |  |  |  |
| C/C [n=300 (71%)] |  | 0.771 | 1.253 | 88777.5 | 0.014 | 0.150 | 0.416 | 941.2 | 1163.1 | 18609.7 | 0.948 | 0.872 | 0.075 | 2.503 | 92.54 | 0.090 | 769.1 |
| C/T [n=90 (21%)] |  | 0.819 | 1.211 | 88761.4 | 0.017 | 0.148 | 0.434 | 934.7 | 1130.6 | 18449.9 | 0.987 | 0.994 | 0.050 | 2.467 | 92.08 | 0.089 | 759.6 |
| T/T [n=33 (8%)] |  | 1.072 | 1.100 | 89069.8 | 0.001 | 0.120 | 0.409 | 983.0 | 1213.4 | 18864.2 | 0.828 | 0.950 | 0.050 | 2.495 | 94.54 | 0.080 | 763.5 |
|  | **P value** | **0.470** | **0.416** | **0.977** | **0.103** | **0.405** | **0.868** | **0.397** | **0.580** | **0.558** | **0.483** | **0.094** | **0.947** | **0.925** | **0.774** | **0.266** | **0.785** |
| *RAD51B* rs61985136 |  |  |  |  |  |  |  |  |  |  |  |  |  |  |  |  |  |
| T/T [n=165 (39%)] |  | 0.837 | 1.179 | 87874.8 | 0.015 | 0.141 | 0.441 | 939.4 | 1141.8 | 18582.9 | 0.925 | 0.889 | 0.050 | 2.497 | 92.45 | 0.085 | 755.4 |
| C/T [n=204 (49%)] |  | 0.792 | 1.300 | 89760.2 | 0.012 | 0.153 | 0.408 | 962.2 | 1169.0 | 18613.4 | 0.977 | 0.924 | 0.072 | 2.513 | 91.25 | 0.090 | 774.3 |
| C/C [n=51 (12%)] |  | 0.771 | 1.105 | 88041.1 | 0.016 | 0.140 | 0.404 | 889.5 | 1178.6 | 18523.9 | 0.901 | 0.875 | 0.099 | 2.465 | 98.56 | 0.094 | 775.8 |
|  | **P value** | **0.934** | **0.074** | **0.045** | **0.192** | **0.572** | **0.526** | **0.031** | **0.756** | **0.955** | **0.642** | **0.714** | **0.939** | **0.919** | **0.025** | **0.170** | **0.261** |
| *LIPC* rs2043085 |  |  |  |  |  |  |  |  |  |  |  |  |  |  |  |  |  |
| T/T [n=159 (38%)] |  | 0.414 | 1.153 | 88157.2 | 0.014 | 0.151 | 0.412 | 942.9 | 1145.3 | 18489.7 | 0.906 | 0.899 | 0.100 | 2.426 | 90.14 | 0.088 | 759.6 |
| C/T [n=189 (45%)] |  | 0.462 | 1.266 | 89392.6 | 0.014 | 0.145 | 0.429 | 939.7 | 1184.3 | 18613.4 | 1.016 | 0.886 | 0.050 | 2.522 | 93.64 | 0.093 | 774.8 |
| C/C [n=74 (17%)] |  | 0.377 | 1.314 | 88664.0 | 0.015 | 0.146 | 0.413 | 953.0 | 1128.7 | 18811.7 | 0.862 | 0.955 | 0.050 | 2.555 | 95.41 | 0.079 | 760.1 |
|  | **P value** | **0.442** | **0.136** | **0.315** | **0.624** | **0.877** | **0.851** | **0.865** | **0.505** | **0.492** | **0.133** | **0.571** | **0.571** | **0.378** | **0.049** | **0.149** | **0.424** |
| *CETP* rs5817082 |  |  |  |  |  |  |  |  |  |  |  |  |  |  |  |  |  |
| C/C [n=264 (63%)] |  | 0.827 | 1.223 | 88880.0 | 0.014 | 0.143 | 0.441 | 932.2 | 1179.7 | 18585.6 | 0.973 | 0.891 | 0.050 | 2.586 | 93.18 | 0.088 | 772.2 |
| CA/C [n=141 (34%)] |  | 0.797 | 1.231 | 88722.4 | 0.015 | 0.151 | 0.385 | 963.9 | 1121.2 | 18518.9 | 0.898 | 0.935 | 0.099 | 2.358 | 91.54 | 0.092 | 752.2 |
| CA/CA [n=14 (3%)] |  | 0.553 | 1.280 | 88566.4 | 0.014 | 0.158 | 0.387 | 963.1 | 1238.1 | 19105.3 | 1.014 | 0.840 | 0.278 | 2.299 | 92.36 | 0.070 | 799.4 |
|  | **P value** | **0.985** | **0.947** | **0.973** | **0.813** | **0.771** | **0.182** | **0.220** | **0.287** | **0.554** | **0.507** | **0.599** | **0.160** | **0.011** | **0.654** | **0.681** | **0.146** |
| *CTRB2/CTRB1* rs55993634* |  |  |  |  |  |  |  |  |  |  |  |  |  |  |  |  |  |
| G/G [n=353 (84%)] |  | 0.764 | 1.233 | 89142.6 | 0.014 | 0.148 | 0.424 | 953.1 | 1158.2 | 18646.4 | 0.939 | 0.915 | 0.050 | 2.491 | 92.98 | 0.090 | 770.1 |
| G/C or C/C [n=68 (16%)] |  | 1.017 | 1.246 | 87106.5 | 0.019 | 0.146 | 0.400 | 885.4 | 1173.5 | 18373.5 | 0.993 | 0.850 | 0.142 | 2.515 | 90.84 | 0.084 | 749.6 |
|  | **P value** | **0.022** | **0.884** | **0.043** | **0.048** | **0.902** | **0.547** | **0.004** | **0.774** | **0.286** | **0.539** | **0.309** | **0.071** | **0.813** | **0.346** | **0.716** | **0.188** |
| *TMEM97/VTN* rs11080055 |  |  |  |  |  |  |  |  |  |  |  |  |  |  |  |  |  |
| A/A [n=118 (28%)] |  | 0.656 | 1.186 | 90113.2 | 0.014 | 0.135 | 0.386 | 961.9 | 1149.8 | 18187.5 | 0.954 | 0.840 | 0.050 | 2.422 | 90.63 | 0.089 | 773.0 |
| A/C [n=194 (47%)] |  | 0.861 | 1.225 | 87845.1 | 0.014 | 0.150 | 0.435 | 939.8 | 1152.3 | 18724.2 | 0.954 | 0.898 | 0.062 | 2.469 | 92.57 | 0.086 | 760.3 |
| C/C [n=105 (25%)] |  | 0.885 | 1.268 | 89152.4 | 0.014 | 0.150 | 0.420 | 933.2 | 1195.9 | 18769.4 | 0.894 | 0.982 | 0.128 | 2.649 | 94.94 | 0.091 | 774.8 |
|  | **P value** | **0.317** | **0.638** | **0.033** | **0.671** | **0.509** | **0.377** | **0.440** | **0.614** | **0.031** | **0.715** | **0.092** | **0.139** | **0.065** | **0.172** | **0.256** | **0.498** |
| *NPLOC4/TSPAN10* rs6565597 |  |  |  |  |  |  |  |  |  |  |  |  |  |  |  |  |  |
| C/C [n=153 (39%)] |  | 0.833 | 1.222 | 88811.5 | 0.015 | 0.138 | 0.429 | 932.0 | 1180.1 | 18581.6 | 0.972 | 0.939 | 0.102 | 2.461 | 92.93 | 0.084 | 765.6 |
| C/T [n=177 (45%)] |  | 0.782 | 1.219 | 89122.2 | 0.013 | 0.157 | 0.439 | 932.8 | 1172.6 | 18644.6 | 0.984 | 0.899 | 0.052 | 2.556 | 91.89 | 0.094 | 762.3 |
| T/T [n=64 (16%)] |  | 0.901 | 1.266 | 89026.4 | 0.014 | 0.123 | 0.368 | 999.6 | 1096.0 | 18387.1 | 0.845 | 0.889 | 0.050 | 2.440 | 94.95 | 0.090 | 785.1 |
|  | **P value** | **0.590** | **0.878** | **0.935** | **0.477** | **0.102** | **0.272** | **0.023** | **0.336** | **0.658** | **0.323** | **0.699** | **0.644** | **0.424** | **0.476** | **0.574** | **0.400** |
| *C3* rs2230199 |  |  |  |  |  |  |  |  |  |  |  |  |  |  |  |  |  |
| C/C [n=276 (66%)] |  | 0.417 | 1.215 | 88943.5 | 0.015 | 0.152 | 0.424 | 945.5 | 1145.3 | 18541.8 | 0.969 | 0.886 | 0.075 | 2.442 | 92.91 | 0.091 | 767.2 |
| C/G [n=125 (30%)] |  | 0.490 | 1.243 | 88672.7 | 0.014 | 0.143 | 0.400 | 948.6 | 1179.2 | 18715.6 | 0.895 | 0.966 | 0.050 | 2.603 | 92.44 | 0.087 | 756.6 |
| G/G [n=20 (4%)] |  | 0.426 | 1.438 | 87581.2 | 0.012 | 0.110 | 0.487 | 871.9 | 1246.2 | 18351.1 | 0.991 | 0.775 | 0.194 | 2.583 | 88.94 | 0.081 | 808.4 |
|  | **P value** | **0.932** | **0.332** | **0.722** | **0.599** | **0.275** | **0.444** | **0.192** | **0.451** | **0.604** | **0.549** | **0.148** | **0.102** | **0.133** | **0.600** | **0.589** | **0.175** |
| *CNN2* rs113772652* |  |  |  |  |  |  |  |  |  |  |  |  |  |  |  |  |  |
| T/T [n=129 (31%)] |  | 0.767 | 1.218 | 87975.1 | 0.013 | 0.144 | 0.418 | 930.6 | 1154.1 | 18382.0 | 0.979 | 0.870 | 0.061 | 2.527 | 91.36 | 0.095 | 759.1 |
| T/C [n=216 (51%)] |  | 0.759 | 1.261 | 88985.4 | 0.016 | 0.141 | 0.401 | 952.6 | 1173.4 | 18557.7 | 0.920 | 0.919 | 0.081 | 2.468 | 92.62 | 0.084 | 767.3 |
| C/C [n=75 (18%)] |  | 0.945 | 1.172 | 89795.9 | 0.010 | 0.165 | 0.474 | 940.6 | 1129.0 | 19056.0 | 0.966 | 0.903 | 0.050 | 2.531 | 94.51 | 0.092 | 782.2 |
|  | **P value** | **0.777** | **0.570** | **0.231** | **0.008** | **0.319** | **0.195** | **0.542** | **0.696** | **0.052** | **0.694** | **0.662** | **0.089** | **0.725** | **0.450** | **0.179** | **0.399** |
| *APOE* rs429358 |  |  |  |  |  |  |  |  |  |  |  |  |  |  |  |  |  |
| T/T [n=325 (79%)] |  | 0.441 | 1.243 | 88849.8 | 0.014 | 0.143 | 0.426 | 944.5 | 1160.7 | 18672.5 | 0.987 | 0.913 | 0.070 | 2.465 | 92.29 | 0.090 | 763.5 |
| T/C or C/C [n=89 (21%)] |  | 0.375 | 1.201 | 88688.9 | 0.016 | 0.161 | 0.400 | 941.2 | 1155.9 | 18279.7 | 0.835 | 0.893 | 0.050 | 2.616 | 93.06 | 0.087 | 772.5 |
|  | **P value** | **0.546** | **0.585** | **0.860** | **0.991** | **0.204** | **0.474** | **0.877** | **0.921** | **0.086** | **0.052** | **0.736** | **0.432** | **0.102** | **0.709** | **0.437** | **0.516** |
| *MMP9* rs253560006 |  |  |  |  |  |  |  |  |  |  |  |  |  |  |  |  |  |
| TTTTC/TTTTC [n=330 (80%)] |  | 0.807 | 1.242 | 88838.6 | 0.014 | 0.147 | 0.422 | 944.1 | 1187.7 | 18585.9 | 0.958 | 0.896 | 0.099 | 2.527 | 92.82 | 0.087 | 770.3 |
| TTTTC/T [n=72 (17%)] |  | 0.823 | 1.155 | 89019.4 | 0.014 | 0.134 | 0.432 | 934.8 | 1088.1 | 18616.6 | 0.952 | 0.976 | 0.050 | 2.415 | 92.48 | 0.098 | 756.7 |
| T/T [n=11 (3%)] |  | 0.990 | 1.451 | 87271.3 | 0.013 | 0.129 | 0.324 | 975.8 | 989.1 | 19128.6 | 0.457 | 0.673 | 0.054 | 2.496 | 92.03 | 0.108 | 726.5 |
|  | **P value** | **0.808** | **0.310** | **0.778** | **0.852** | **0.639** | **0.537** | **0.769** | **0.053** | **0.659** | **0.075** | **0.128** | **0.245** | **0.531** | **0.978** | **0.430** | **0.351** |
| *C20orf85* rs117420707* |  |  |  |  |  |  |  |  |  |  |  |  |  |  |  |  |  |
| T/T [n=383 (91%)] |  | 0.804 | 1.223 | 88652.2 | 0.014 | 0.148 | 0.423 | 943.0 | 1158.9 | 18566.7 | 0.945 | 0.901 | 0.061 | 2.494 | 92.64 | 0.088 | 768.7 |
| T/TA or TA/TA [n=39 (9%)] |  | 0.813 | 1.321 | 90247.9 | 0.018 | 0.144 | 0.390 | 946.1 | 1151.1 | 18877.0 | 0.964 | 0.945 | 0.081 | 2.522 | 91.96 | 0.098 | 746.7 |
|  | **P value** | **0.831** | **0.372** | **0.211** | **0.291** | **0.863** | **0.525** | **0.919** | **0.907** | **0.340** | **0.865** | **0.585** | **0.978** | **0.834** | **0.812** | **0.200** | **0.264** |
| *SYN3/TIMP3* rs5754227 |  |  |  |  |  |  |  |  |  |  |  |  |  |  |  |  |  |
| T/T [n=325 (78%)] |  | 0.441 | 1.228 | 89259.1 | 0.013 | 0.148 | 0.419 | 948.9 | 1137.4 | 18664.2 | 0.937 | 0.911 | 0.053 | 2.531 | 92.66 | 0.087 | 765.1 |
| T/C [n=85 (20%)] |  | 0.408 | 1.261 | 87374.1 | 0.017 | 0.141 | 0.417 | 929.3 | 1244.6 | 18245.0 | 0.944 | 0.870 | 0.102 | 2.402 | 93.06 | 0.094 | 768.8 |
| C/C [n=10 (2%)] |  | 0.471 | 1.148 | 87236.9 | 0.012 | 0.120 | 0.433 | 903.6 | 1293.2 | 19020.4 | 1.209 | 0.942 | 0.244 | 2.200 | 90.75 | 0.104 | 810.6 |
|  | **P value** | **0.603** | **0.843** | **0.100** | **0.185** | **0.668** | **0.987** | **0.520** | **0.053** | **0.159** | **0.428** | **0.758** | **0.559** | **0.182** | **0.918** | **0.354** | **0.479** |
| *SLC16A8* rs8135665 |  |  |  |  |  |  |  |  |  |  |  |  |  |  |  |  |  |
| C/C [n=268 (65%)] |  | 0.839 | 1.224 | 89252.6 | 0.015 | 0.153 | 0.432 | 931.1 | 1153.9 | 18577.4 | 1.000 | 0.900 | 0.061 | 2.500 | 92.42 | 0.089 | 769.6 |
| C/T [n=116 (28%)] |  | 0.699 | 1.200 | 88476.2 | 0.013 | 0.136 | 0.404 | 964.5 | 1198.4 | 18688.4 | 0.866 | 0.938 | 0.050 | 2.490 | 93.55 | 0.089 | 768.4 |
| T/T [n=30 (7%)] |  | 0.920 | 1.388 | 87448.2 | 0.013 | 0.135 | 0.369 | 956.8 | 1111.8 | 18698.2 | 0.794 | 0.855 | 0.108 | 2.529 | 91.46 | 0.086 | 762.0 |
|  | **P value** | **0.731** | **0.356** | **0.363** | **0.499** | **0.404** | **0.451** | **0.222** | **0.466** | **0.850** | **0.072** | **0.643** | **0.122** | **0.970** | **0.775** | **0.834** | **0.945** |

Analyses are based on ANOVA t-tests for normally distributed trace elements, and Kruskal-Wallis H tests for non-normally distributed trace elements.
None of the trace-elements achieved the threshold for statistical significance after applying Bonferroni correction for 16 covariates (the Bonferroni adjusted threshold for statistical significance was defined as P<0.003). *The following alternative variants were used instead of the original discovery variants: *ACAD10/BRAP*: rs61941272 for rs61941274 (R^2^ 1.0), *C20orf85*: rs117420707 for rs201459901 (R^2^ 0.985), *CNN2*: rs113772652 for rs67538026 (R^2^ 0.996), *CTRB2/CTRB1*: rs55993634 for rs72802342 (R^2^ 0.889), *PRLR/SPEF2*: rs74767144 for rs114092250 (R^2^ 0.773). Abbreviations: ANOVA, analysis of variance; As, arsenic; Ba, barium; Ca, calcium; Cd, cadmium; Co, cobalt; Cr, chromium; Cu, copper; Fe, iron; Mg, magnesium; Mn, manganese; Mo, molybdenum; nAMD, neovascular age-related macular degeneration; Pb, lead; Sb, antimony; Se, selenium; V, vanadium; Zn, zinc
